# Tying Covalent Organic Frameworks through Alkene Metathesis and Supported Platinum as Efficient Catalysts for Hydrosilylation

**DOI:** 10.3390/nano12030499

**Published:** 2022-01-31

**Authors:** Defa Gu, Guangwen Li, Yushan Liu, Yuzhou Liu

**Affiliations:** 1 School of Chemistry, Beihang University, Bejing 100191, China; gudefa1993@163.com; 2 Research Institute of Petroleum Processing, SINOPEC, Beijing 100083, China; liguangwen.ripp@sinopec.com; 3 Trinity School of Durham and Chapel Hill, Durham, NC 27705, USA; 13611017779@163.com; 4 Advanced Innovation Center for Biomedical Engineering, Beihang University, Beijing 100191, China

**Keywords:** hydrolytic stability, hierarchical bond formation, alkene metathesis, hydrosilylation

## Abstract

Recently there has been a great interest in covalent organic frameworks due to their fascinating properties. Current approaches to improve their hydrolytic stability mainly rely on the transformation of the dynamic bonds into strong and irreversible bonds, but these approaches also reduce the versatility of the frameworks. Herein, we would like to demonstrate a solution to this dilemma by forming hierarchical bonds through olefin metathesis to produce highly stable COFs. Our approach allows unprecedented opportunities for post-modification of the inner space through the dynamic imine bonds while maintaining the integrity of the framework. Specifically, we demonstrate an amorphous-to-crystalline transformation. In addition, the porosity can be enhanced by up to 70% after full removal of the amine subunits. Overall, our work provides a new direction for the generation of highly stable while still versatile COFs. Meanwhile, platinum(II) complexes can be supported on BHU-2 (Pt@BHU-2) or BHU-2-Oxidate(Pt@BHU-2-Oxidate) as efficient catalysts for hydrosilylation.

## 1. Introduction

Covalent organic frameworks (COFs) are crystalline organic networks, the formation of which are mainly achieved through dynamic covalent bonds. The easy synthesis and unique properties of COFs have been consistently attracting people’s attention, and they have remained in the focus of intensive investigations. A deeper understanding of the growth mechanism has led to precise control of the morphology and porosity, and the availability of many well-designed COFs tailored to specific applications has promoted their wide adoption in many other areas [1,2,3,4,5,6,7,8,9,10,11]. Meanwhile, the low hydrolytic stability of most COFs has remained a critical issue for these applications. Significant efforts have been made to stabilize COFs, and previous reports have emphasized the modification of the nature of these dynamic bonds by transforming them into strong and irreversible covalent bonds and hydrogen bonds [12,13,14,15,16,17,18].

Herein, we proposed a new way of generating stabilized COFs through hierarchical bond formation. First, the COF was formed through common dynamic imine bonds. After the COF formation, there is the subsequent formation of strong covalent bonds through pre-installed functional groups was triggered by a second stimulus, for example, the diffusion of catalyst through the pores of the COF. For this purpose, we designed and synthesized a *C_3_* symmetric aldehyde molecule with two kinds of orthogonally reactive groups, aldehyde, and terminal alkene. The aldehyde group reacted with *C_3_* symmetric amine compounds to generate a crystalline COF, and subsequently, the alkene groups were cross-linked through metathesis reaction catalyzed by the diffused Grubbs II catalyst to firmly tie up the overall framework. A similar method was also reflected in a previous article [3]. With this strategy, a highly stable COF was obtained. The resulting COF exhibited significantly higher stability than the untied one, and the tied framework allowed further manipulation of the imine bonds, which led to interesting crystallinity recovery and porosity enhancement. Our work provided a new way of achieving both high stability and high versatility for COFs. Meanwhile, we reported new heterogeneous catalysts consisting of Pt(II) and BHU-2 (Pt@BHU-2) or BHU-2-Oxidate(Pt@BHU-2-Oxidate). These compounds are able to effectively catalyze hydrosilylation.

## 2. Materials and Methods

### 2.1. Chemicals 

Unless otherwise mentioned, all chemicals were purchased through InnoChem, Inc. China. THF and Et_2_O were distilled from Na/benzophenone. Reactions were monitored using analytical thin-layer chromatography. Flash chromatography was performed using silica gel (200–300 mesh).

### 2.2. Characterization and Measurements 

Proton nuclear magnetic resonance (^1^H-NMR) spectra were recorded on Bruker Avance 300 and 400 MHz (Zurich, Switzerland) spectrometers using DSMO-*d*_6_ or CDCl_3_ as the solvent. The ^1^H-NMR were referenced to residual solvent signals at 7.26 ppm (CHCl_3_) or 2.5 ppm (DMSO). Solid-state ^13^C-NMR spectra were collected on a JNM-ECZ600R (Akishima, Tokyo, Japan). The matrix-assisted laser desorption ionization time-of-flight (MALDI-TOF) mass spectrum was collected on a Bruker Autoflex III (Karlsruhe, Germany) at Tsinghua University. Gas adsorption isotherm was measured on a Quadrasorb SI-MP (Florida, FI, USA) surface area analyzer. FTIR spectra were collected using a Thermo Nicolet Nexus470 FTIR instrument. The crystalline phase structure of all samples were observed on a Bruker-D8-Focus powder diffractometer with (Cu Kα irradiation λ = 0.15406 nm) in the range of 2 to 40° (2θ). Inductively coupled plasma mass spectrometry (ICP-MS) was collected on an Aglient 7800. GC-MS was collected on a SHIMADZU GCMS-QP2010 SE (Kyoto, Japan). The morphology and composition of samples were characterized on a field emission scanning electron microscope (FE-SEM, JEOL JSM-7500F, Akishima, Japan) coupled with an energy-dispersive X-ray spectroscope (EDS).

### 2.3. Synthesis of Compound L1

**Scheme 1:**
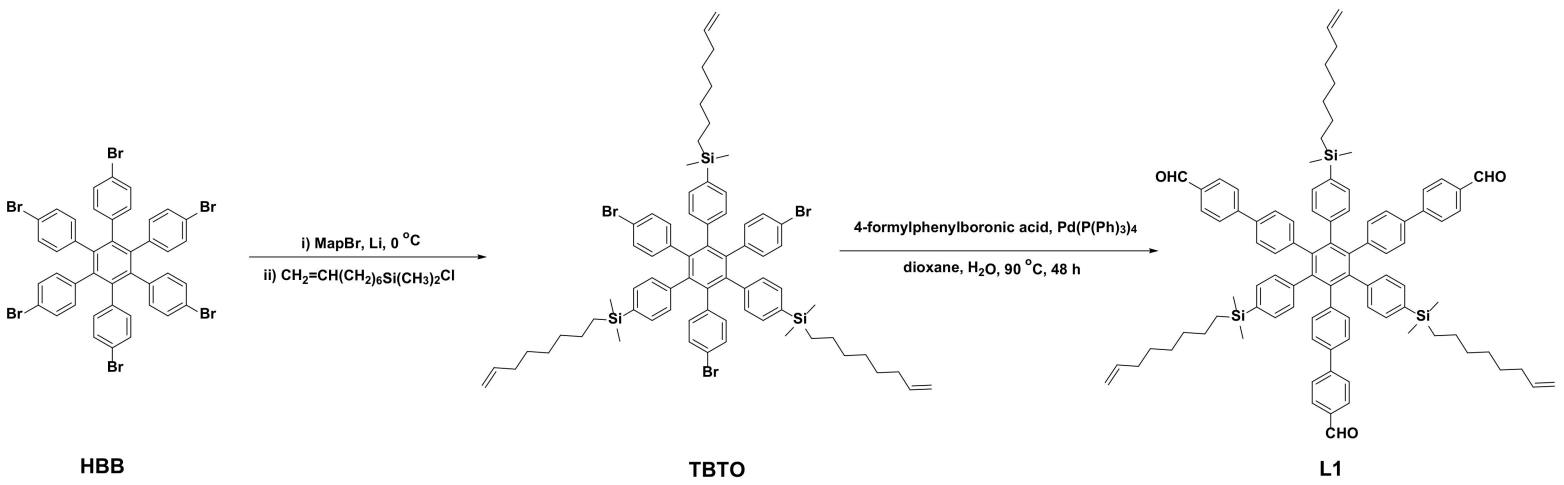
Synthesis of *C_3_* symmetrical aldehyde L1 with pedant alkene groups.

TBTO was synthesized according to our group’s reported method [19]. TBTO (1021 mg, 0.8 mmol) and 4-formylphenylboronic acid were dissolved in 40 mL dioxane, then H_2_O (8 mL) and K_2_CO_3_ (1194 mg, 8.64 mmol) were added. After degassing by argon bubbling, tetrakis(triphenylphosphine)palladium (73.9 mg, 0.064 mmol) were added. The reaction mixture was heated to 90 °C for 48 h. The crude product was purified by column chromatography (petroleum ether/dichloromethane = 2/3) to yield L1 (649 mg, 60%) as a white solid (Scheme 1).

### 2.4. Synthesis of BHU-1

**Scheme 2:**
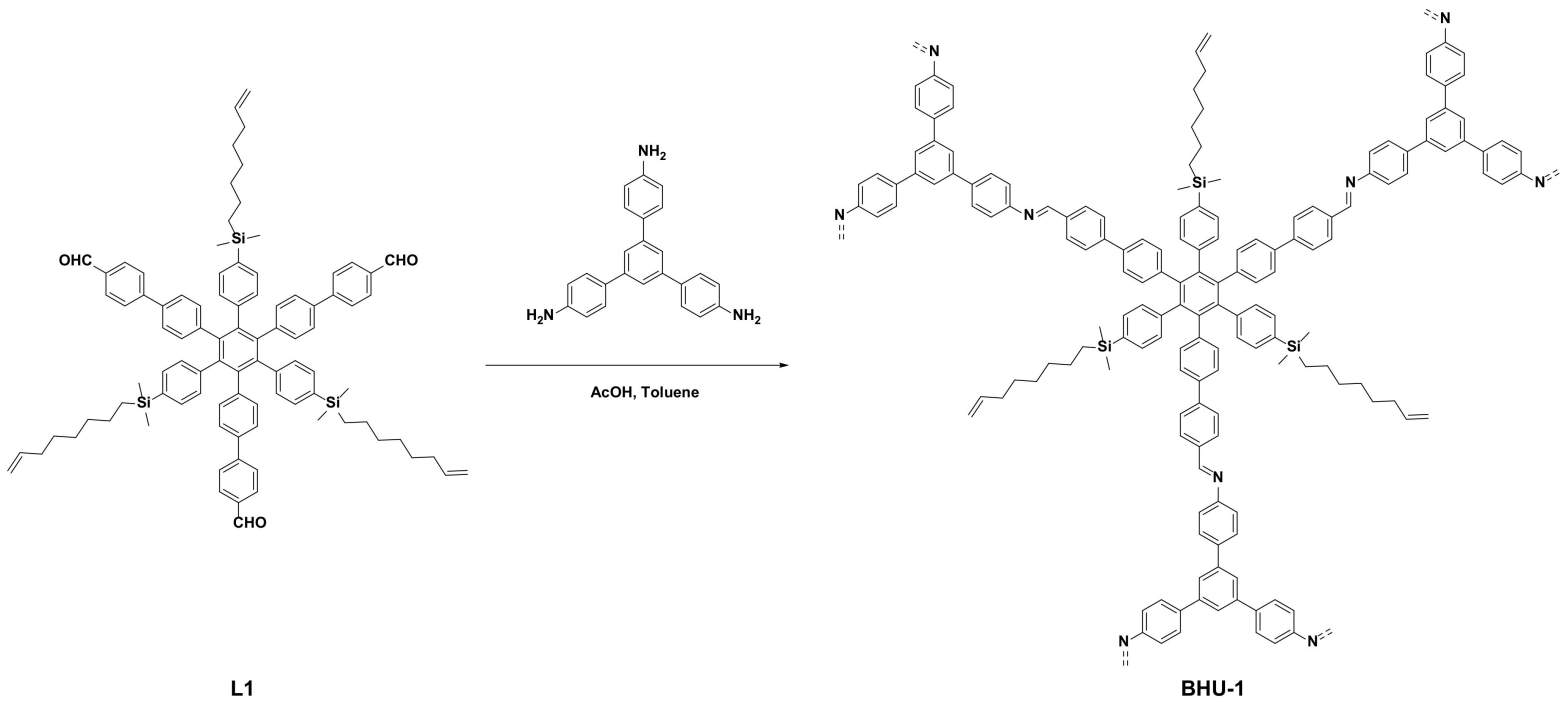
Synthesis of BHU-1.

A 10 mL Teflon reactor was charged with compound L1 (40.5 mg, 0.03 mmol), 1,3,5-tri(4-aminophenyl)benzene (10.5 mg, 0.03 mmol) and toluene (2.7 mL). Then the resulting suspension was sonicated for 5 min. Aqueous AcOH (3 M) (0.09 mL, 0.27 mmol) was added. The mixture was degassed for 10 min by argon and then heated at 120 °C for 10 days. The precipitate was then collected as a yellow solid (45 mg, 90%) (Scheme 2).

### 2.5. Synthesis of BHU-1-T

**Scheme 3:**
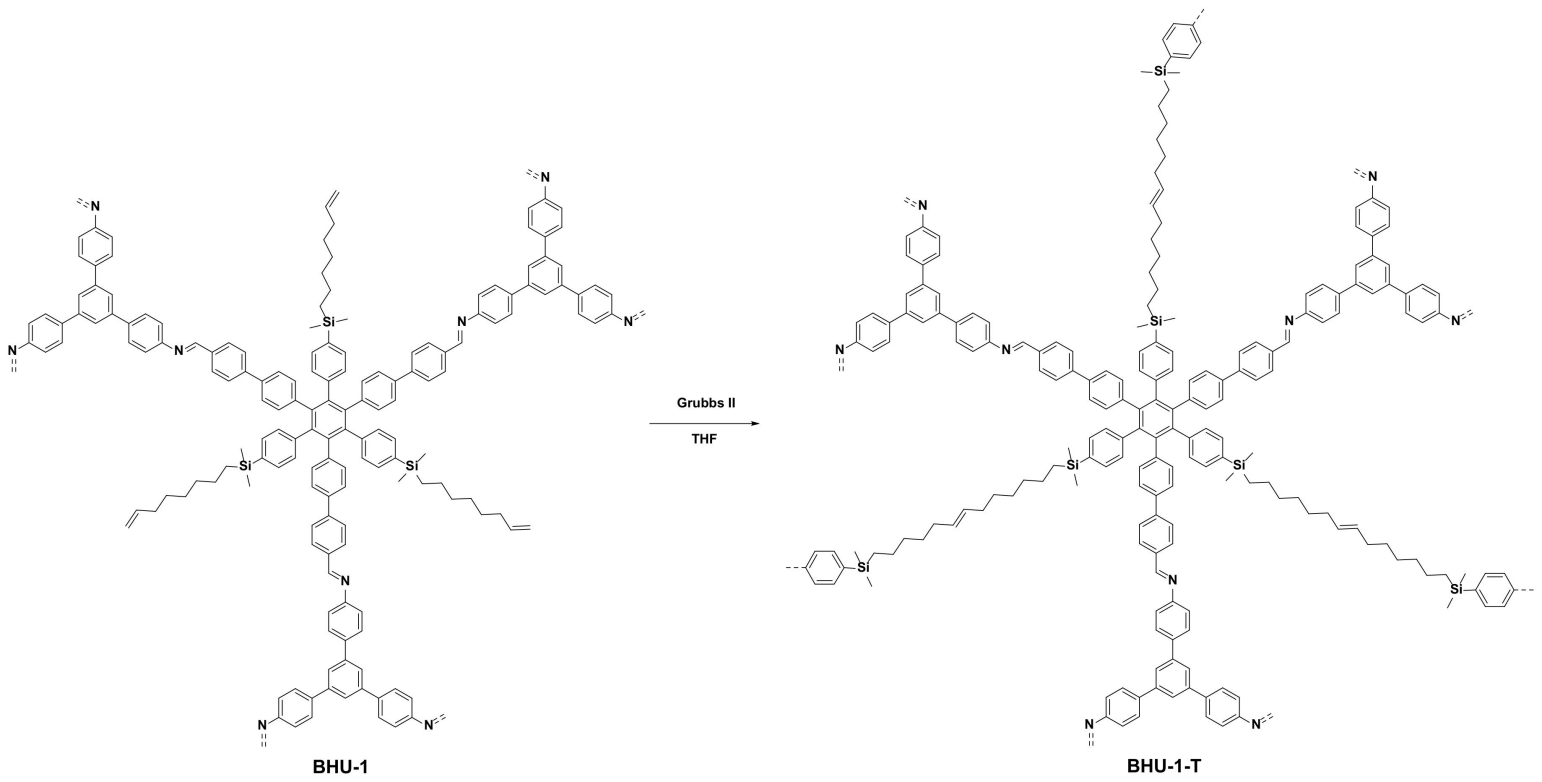
Synthesis of BHU-1-T.

To a three-necked round-bottom flask with a magnetic stir bar, BHU-1 (40 mg) and THF 40 mL were added. The flask was purged three times with argon gas. Grubbs II catalyst (4 mg, 4.6×10 ^− 3^ mmol) was added. The reaction solution was then stirred at 50 °C for two weeks. The product (BHU-1-T) was soaked in THF for 48 h, repeatedly washed with THF, filtrated, and dried overnight under vacuum to obtain a faint yellow solid (38 mg, 95%) (Scheme 3).

### 2.6. Synthesis of BHU-2

**Scheme 4:**
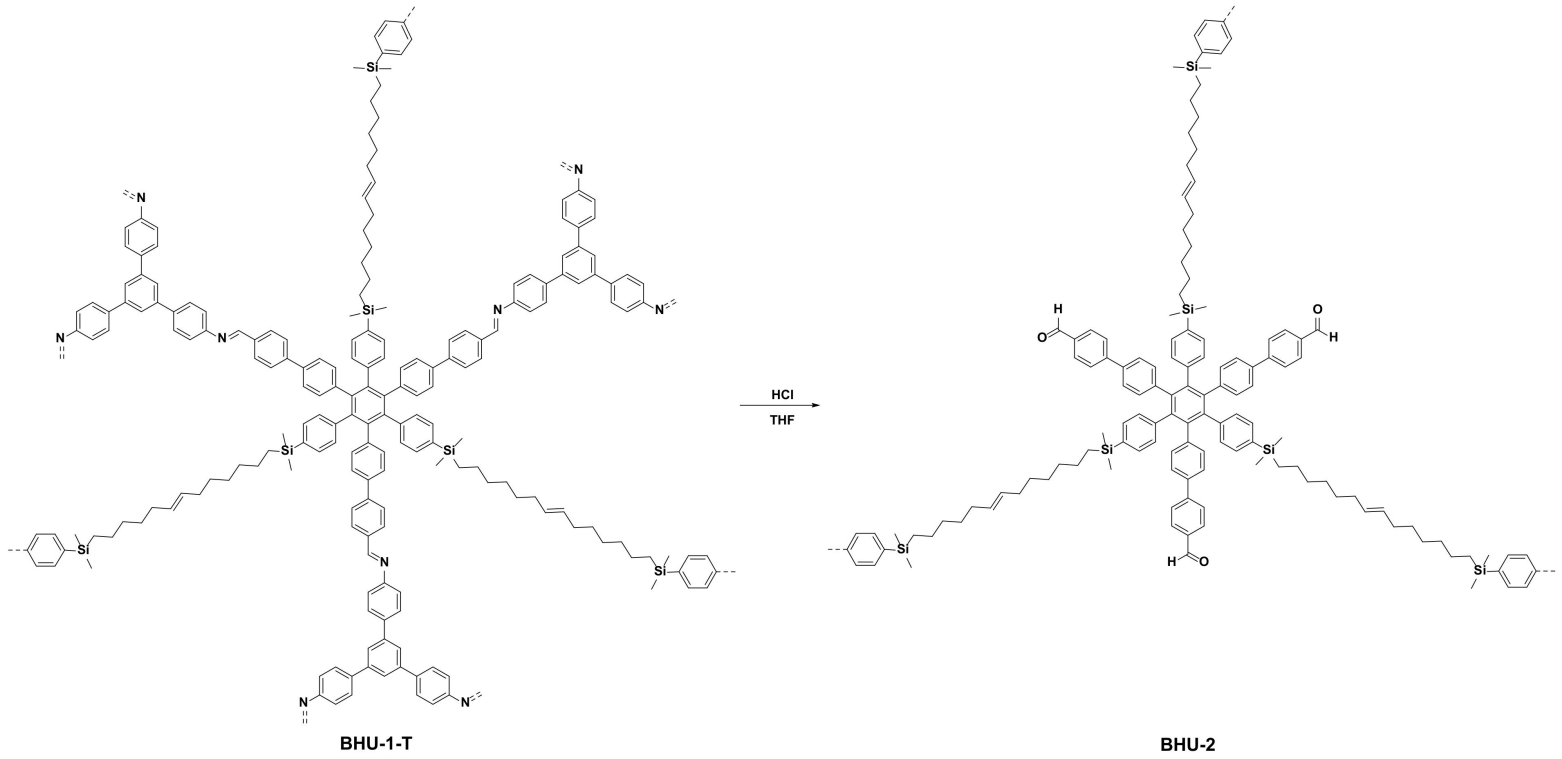
Synthesis of BHU-2.

A total of 5 mg BHU-1-T was soaked in the mixture of 1 mL aqueous HCl (5 M) and 9 mL THF solution for 16 h at room temperature. The product (BHU-2) was collected by filtration and dried overnight under vacuum as a gray solid (3.6 mg, 90%) (Scheme 4).

### 2.7. Synthesis of BHU-2-Oxidate

**Scheme 5:**
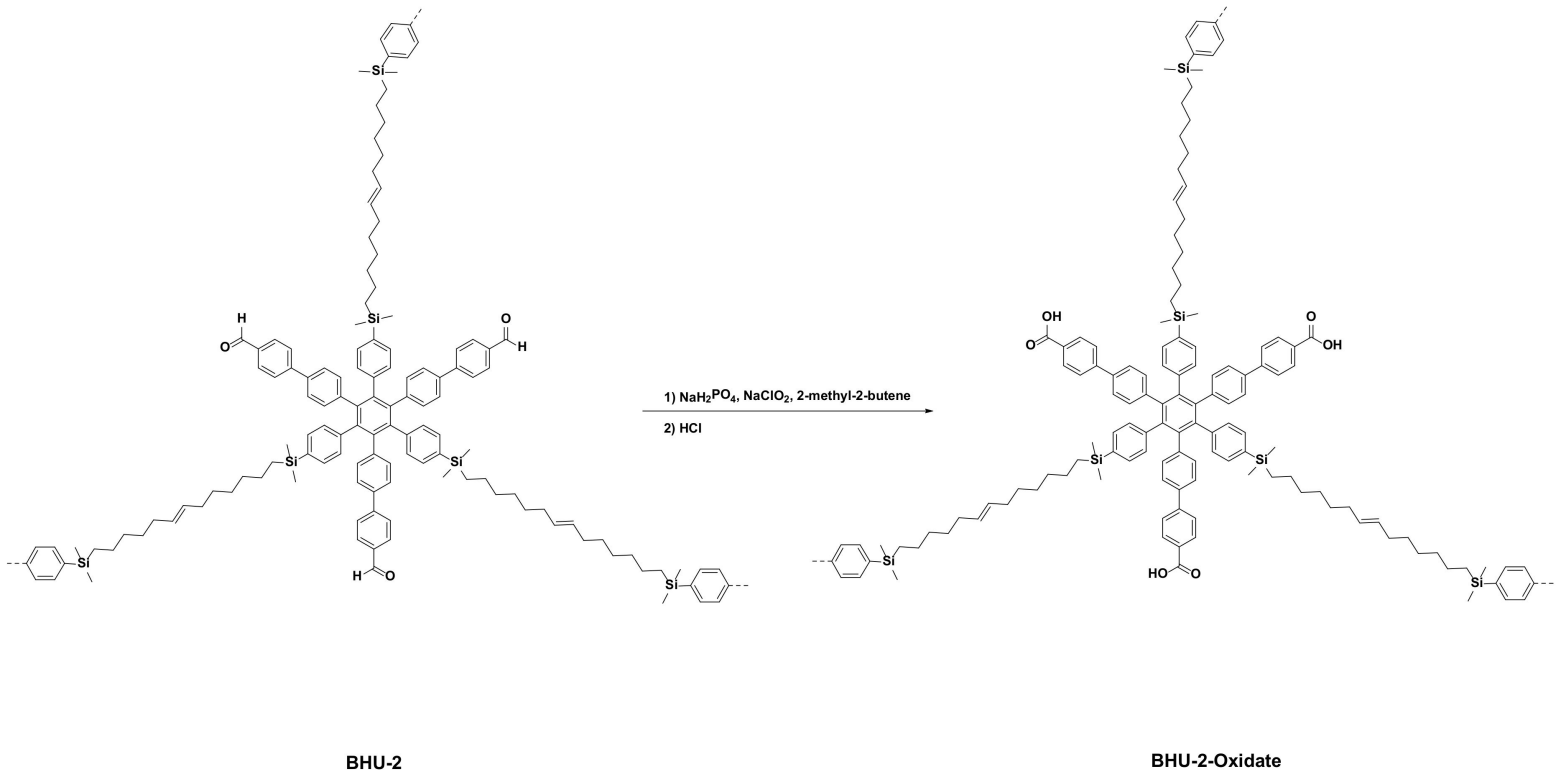
Synthesis of BHU-2-Oxidate.

Solid NaH_2_PO_4_ (90 mg, 0.735 mmol) was added to a solution of BHU-2 (1 mg) in a mixture of THF (0.50 mL), H_2_O (0.10 mL), and 2-methyl-2-butene (0.10 mL), followed by the addition of NaClO_2_ (85 mg, 0.752 mmol). The reaction mixture was impregnated for 5 days. Then solid was collected by filtration and impregnated with 5% HCl (2 mL) for 14 h. The product (BHU-2-Oxidate) was collected by filtration, washed by ethanol, and distilled water. Then the product was dried overnight under vacuum as a brown solid (0.76 mg) (Scheme 5).

### 2.8. Synthesis of Pt@BHU-2 or Pt@BHU-2-Oxidate

Pt@BHU-2 or Pt@BHU-2-Oxidate was added to a solution of Na_2_PtCl_4_ (1 mg) in ethanol (0.10 mL). The reaction mixture was impregnated for 43 h. The product (Pt@BHU-2 or Pt@BHU-2-Oxidate) was collected by filtration, washed with ethanol, and dried overnight under vacuum as a brown solid.

### 2.9. Catalytic Activity Test

To a 5 mL glass bottle, styrene (10.42 mg, 0.1 mmol), dimethylphenylsilane (27.25 mg, 0.2 mmol), catalyst (1 mg), and toluene (1 mL) were added. Then *n*-dodecane (17.03 mg, 0.1 mmol) as the internal standard was added into the mixture. The reaction solution was heated to 60 °C for 8 h. Products were analyzed with GC-MS. Both the conversion and selectivity were obtained through the internal standard method according to the GC-MS data.

The recyclability of catalyst was evaluated in terms of the conversion performance on repeated use for several cycles. Briefly, styrene (10.42 mg, 0.1 mmol), dimethylphenylsilane (27.25 mg, 0.2 mmol), catalyst (1 mg), *n*-dodecane (17.03 mg, 0.1 mmol), and toluene (1 mL) were added. Then the reaction was allowed to proceed at 60 °C for 8 h. The catalyst was collected via filtration, washed with dichloromethane, and dried at room temperature for 12 h.

## 3. Results

### 3.1. Overall Design

**Figure 1:**
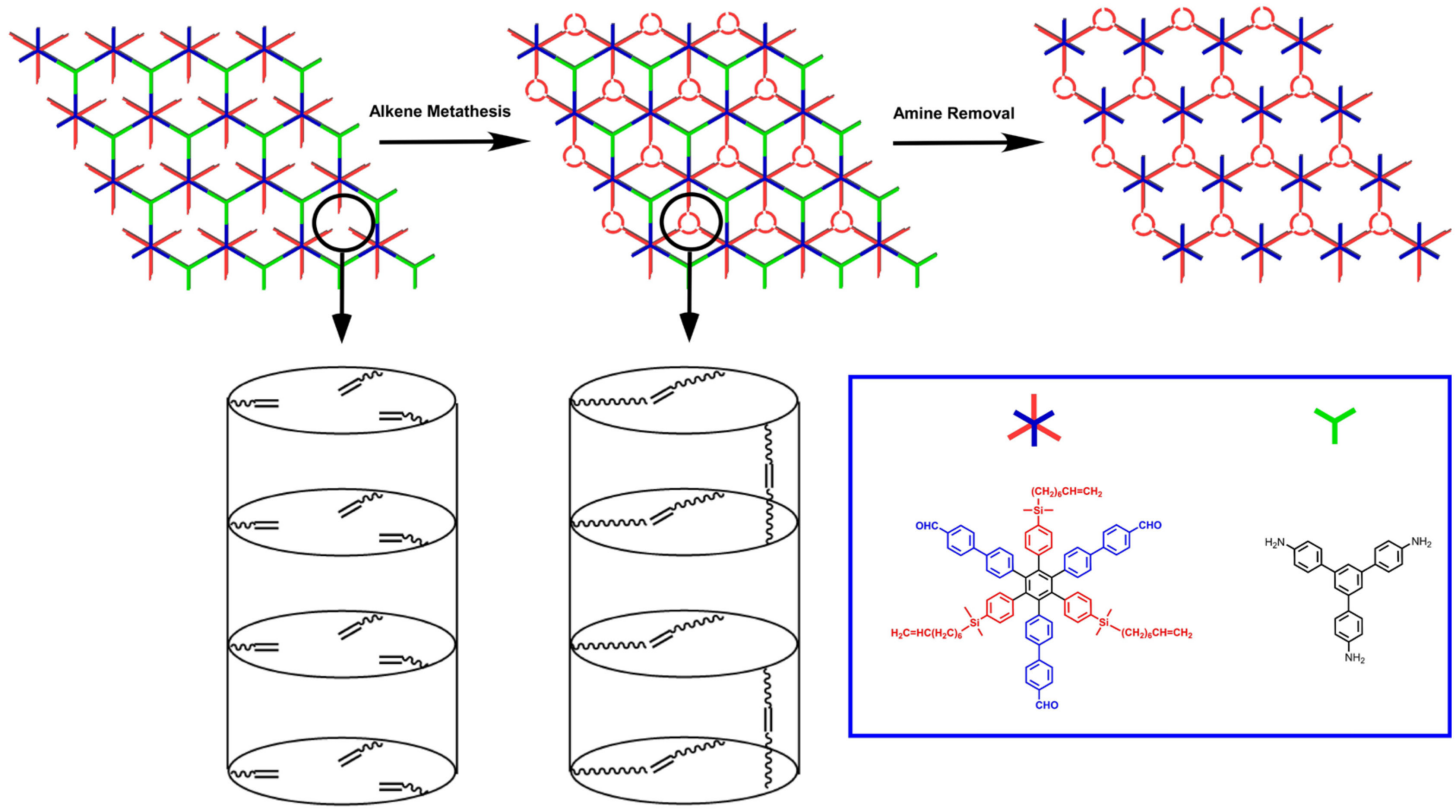
The overall design for hierarchical bond formation within formed COF for the purpose of improving the hydrolytic stability.

Our design for hierarchical bond formation, namely the imine bond formation and alkene metathesis reaction, to obtain stable COF are shown in Figure 1. It was reasoned that the installation of additional alkene groups on the aldehyde precursors could provide a way for a subsequent cross-linking event through alkene metathesis between the aldehyde subunits after the formation of COFs. Special requirements had to be met for this purpose with respect to the length of the alkyl groups connecting the alkenes and the core. The length should be long enough so that alkenes from adjacent cores could be in contact with each other for reaction to occur after the framework is constructed, while it should be not too long to avoid occupying too much free space in the framework and hindering the diffusion of the complex decomposition catalyst. 

Another factor that has to be taken into consideration during the design is the size of the channel. Grubbs II catalyst, which has been a common alkene metathesis catalyst, has a dimension around 1.2 nm by its largest length, and therefore the diameter of the channel of formed COF has to be at least 1.2 nm taking the Van der Waals radius of the framework into consideration.

### 3.2. Synthesis of C_3_ Symmetrical Aldehyde Precursor L1

The above consideration about metathesis reaction was then condensed into the design criteria and choices of suitable precursors to construct COFs. First, as stated above, suitable *C_3_*-symmetrical precursors had to possess both alkene groups and aldehyde groups. The targeted trialdehyde (L1) was synthesized by thermodynamically controlled halogen dancing as the first step to obtain the alternatively lithiated intermediate [20,21]. The alkene groups were then installed efficiently through termination reaction by relative alkene chlorosilane. Aldehyde groups were then introduced through the common Suzuki reaction catalyzed by Pd(PPh_3_)_4_. Finally, L1 was obtained as a white powder with an overall yield of around 60%. As shown in Figure S1, the controlled halogen dancing reaction effectively inhibited the formation of 1,2,4 isomer during the reaction pathway, and only the 1,3,5 product was obtained. The purity of the trialdehyde promises high chances for phase purity of formed COFs.

### 3.3. Preparation and Characterization of BHU-1

**Figure 2:**
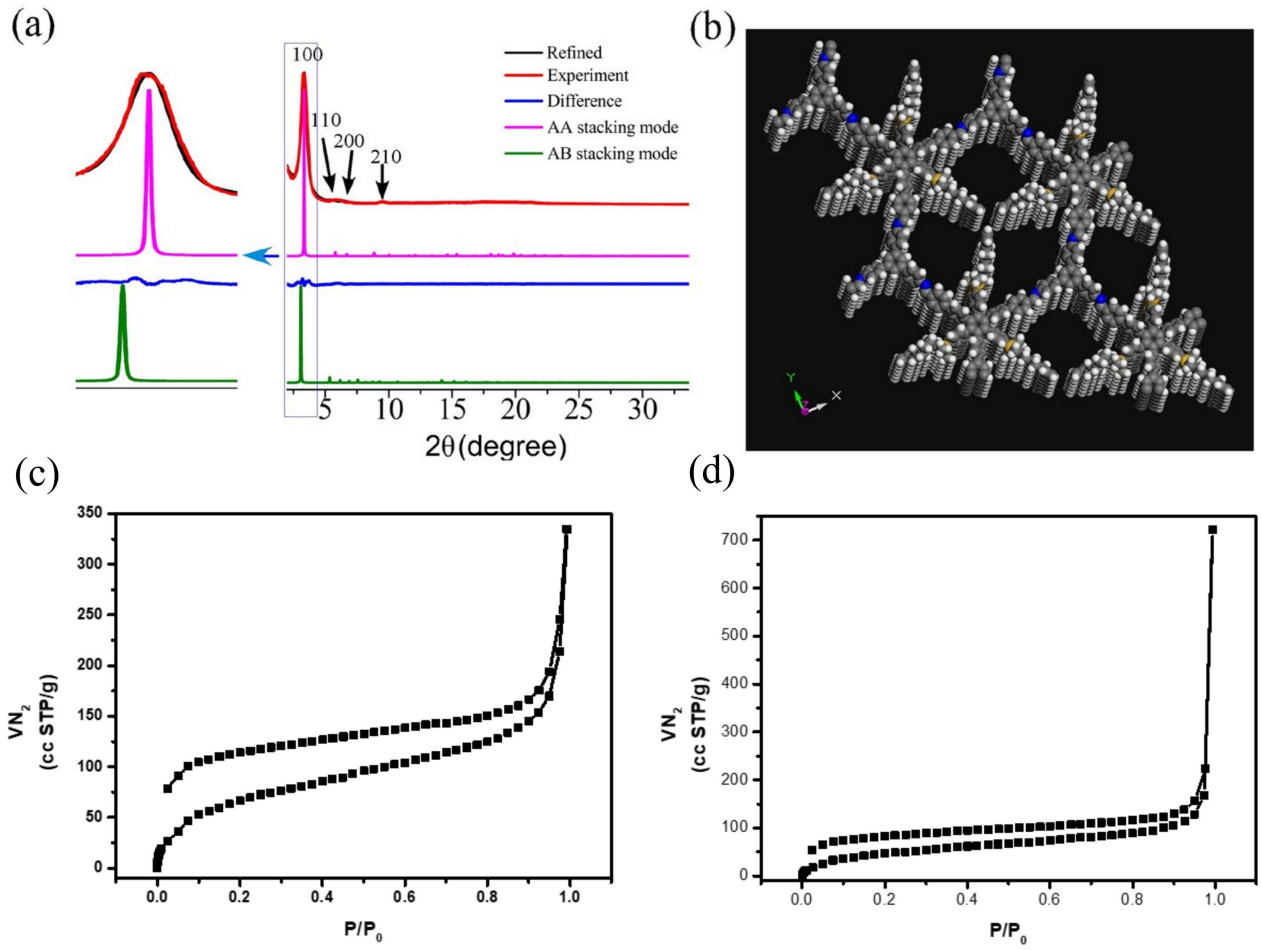
(**a**) Observed PXRD pattern (black curve, the major reflections are assigned) and Pawley refined profile (red curve) (Pawley refinements gave optimized parameters (a = b = 36.40 Å and c = 9.21 Å)), which provided good agreement factors (Rp = 6.46% and Rwp = 8.98%). Difference plot between the observed and refined PXRD patterns (blue curve). Simulated PXRD pattern for AA stacking (eclipsed structure) (pink curve) and AB stacking (green curve) of BHU-1. (**b**) Eclipsed structure of BHU-1 with the unit cell axis indicated. The alkyl chains are fully expanded to just show that they can reach each other for metathesis reaction to happen, but they can also coil within the channels. (**c**) Nitrogen adsorption–desorption isotherms of BHU-1 and (**d**) BHU-1-T.

As shown in Scheme 2, upon reacting L1 (40.5 mg, 0.03 mmol) with 1,3,5-tri(4-aminophenyl)benzene (10.5 mg, 0.03 mmol) in 2.7 mL toluene with 3 M acetic acid (AcOH) (0.09 mL, 0.27 mmol) at 120 °C for 10 days, BHU-1 was formed as a yellow powder. The detailed structure of BHU-1 was determined by comparing its powder X-ray diffraction (PXRD) pattern with a simulated structural model (Figure 2a), in which the hexaphenylbenzene subunits stack on top of each other to form pillars (AA stacking) connected by imine bonds to generate a 2D layered structure (Figure 2b). The geometry and the unit cell of the model were optimized to a local energy minimum before the diffraction pattern was simulated. The eclipsed stacking (AA) and staggered stacking (AB) structures were evaluated (see Supporting Information S5 for details). As shown in Figure 2a, PXRD of BHU-1 revealed a strong peak at 2ϴ =3.32^o^, and this corresponded to the (100) reflections due to the periodicity of the covalent network. The peaks at 5.8° and 9.3° were related to the (110) and (300) respectively. Hexaphenylbenzene derivatives have been known to mostly exhibit such stacking model in solid-state due to the efficient packing between the phenyl propellers [22,23]. The vertical stacking led to the generation of permanent porosity, which was estimated by nitrogen adsorption isotherms as shown in Figure 2c and Figure S2. The BET surface was estimated to be around 259 m^2^·g^−^^1^. The main pore diameter was around 1.4 nm. As shown in Figure S3, the offset model (AB stacking) was also constructed, but apparently, the powder diffraction pattern of AB stacking did not match with the experimental patterns, with the major (100) peak offset by 0.23 degrees as shown in Figure 2a.

### 3.4. Tying of BHU-1

**Figure 3:**
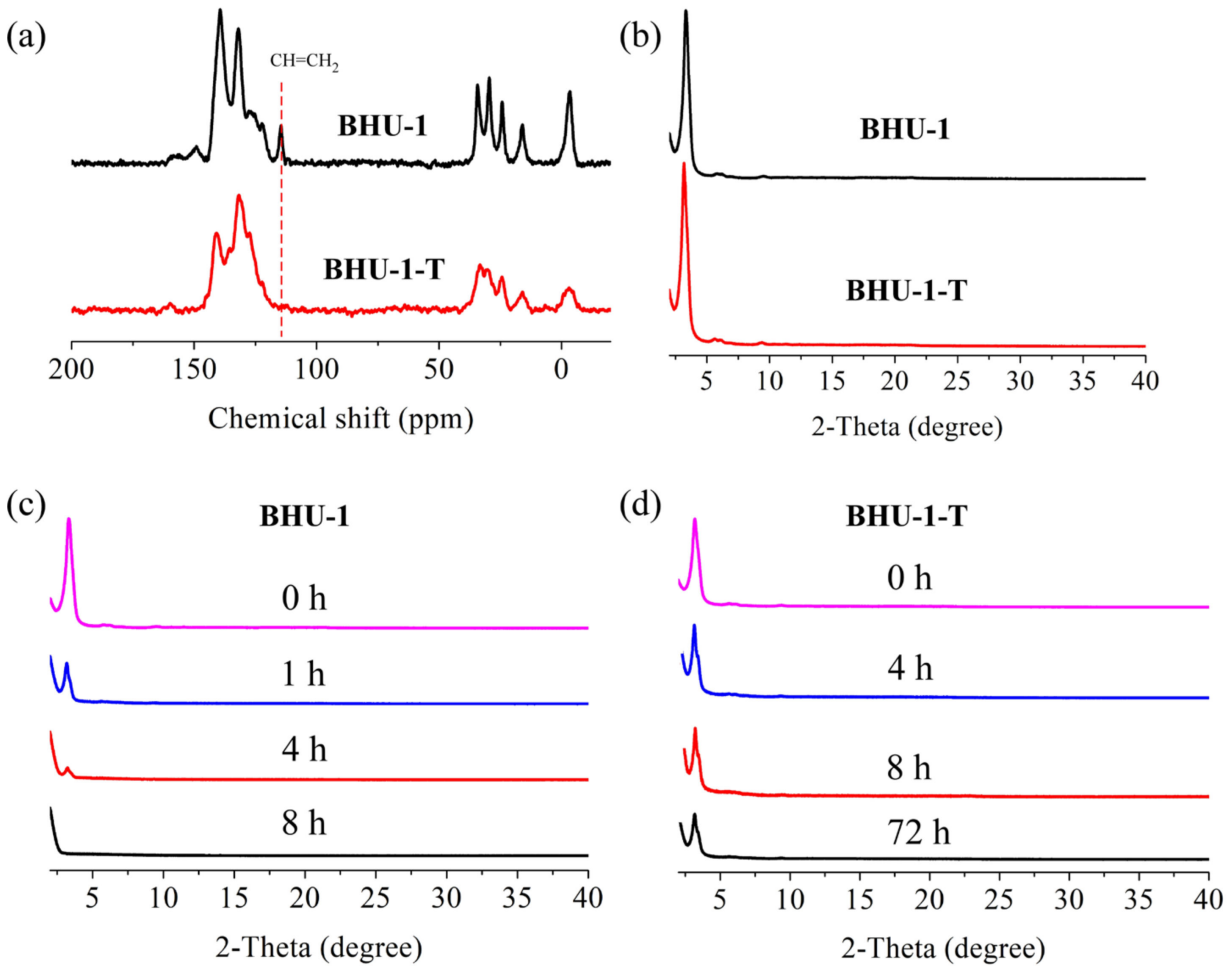
(**a**) ^13^C CP-MAS solid-state NMR spectrum of BHU-1 and BHU-1-T for the identification of conversion of alkene groups through alkene metathesis. (**b**) PXRD patterns of BHU-1 and BHU-1-T. PXRD patterns of BHU-1 (**c**) and BHU-1-T (**d**) after treatment under acid conditions (in the mixture of 1 mL aqueous HCl (1 M) and 9 mL THF).

The introduction of long alkyl chains with terminal alkenes allowed further metathesis reaction to tie up the COF. BHU-1 (40 mg) was soaked in a 40 mL dry tetrahydrofuran (THF) solution of Grubbs II catalyst (4 mg, 4.6 × 10 ^−3^ mmol) for two weeks at 50 °C. After the reaction, the precipitate was soaked in THF for 48 h and repeatedly washed with THF to remove the Grubbs catalyst, and then was isolated by filtration to obtain tied BHU-1-T, which was then washed extensively by THF in order to remove any residual catalyst. Inductively coupled plasma mass spectrometry (ICP-MS) showed that only 0.36 ppm Ru was present after washing. BHU-1-T was a network structure formed by the connection of imine bonds and C=C double bonds. We also conducted solid-state ^13^C-NMR for the washed BHU-1-T, which revealed the high efficiency of the tying process. As shown in Figure 3a, the 114 ppm peak corresponded to terminal alkene molecules in BHU-1, and the peak fully disappeared in BHU-1-T due to the formation of internal alkenes. Moreover, the characteristic peak of 1006 cm^−1^ for R-C=C-R bonds appeared in the IR spectroscopy of BHU-1-T (Figure S4a). The high efficiency of alkene metathesis reaction in BHU-1-T was not unexpected due to the large volume inside BHU-1, and this was consistent with previous reports of such reactions in confined space [24,25]. The BET surface was estimated to be around 179 m^2^·g^−1^ and the main pore diameter was around 1.4 nm (Figure 2d and Figure S5).

As shown in Figure 3b, BHU-1-T exhibited an almost identical diffraction pattern as BHU-1 and therefore the overall crystallinity was maintained during the tying process. In addition, BHU-1-T exhibited remarkably higher stability than BHU-1. As shown in Figure 3c, 3d, BHU-1 and BHU-1-T (5 mg each) were both soaked in the mixture of 1 mL aqueous HCl (1 M) and 9 mL THF, and then it was found that BHU-1 completely lost its crystallinity after 8 h, while BHU-1-T maintained most of its crystallinity even after 72 h. The loss of mass of BHU-1-T after hydrolytic treatment was 4.8%. With the introduction of hydrophobic groups in the pore wall, the stability of the skeleton is improved. This is attributed to the kinetic blocking that effectively protects the hydrolytically susceptible backbone. A similar phenomenon has been reported in a previous article [26]. The results of TGA showed that the loss of mass of BHU-1 and BHU-1-T was 44.42% and 39.78% respectively (Figure S6). Based on the above results, we could conclude that BHU-1-T becomes more stable after tying.

The distinguishable feature of our approach to preparing stable COF is that the stabilizing effect is not due to the structural enforcement of the dynamic imine bonds as other approaches but the hierarchical construction of different kinds of covalent bonds within the COF. Given the reversible nature of the dynamic imine bonds, we then proceeded to examine the possibility of manipulating them and therefore the inner free space within BHU-1-T, which was almost impossible for other stable COFs. 

**Figure 4:**
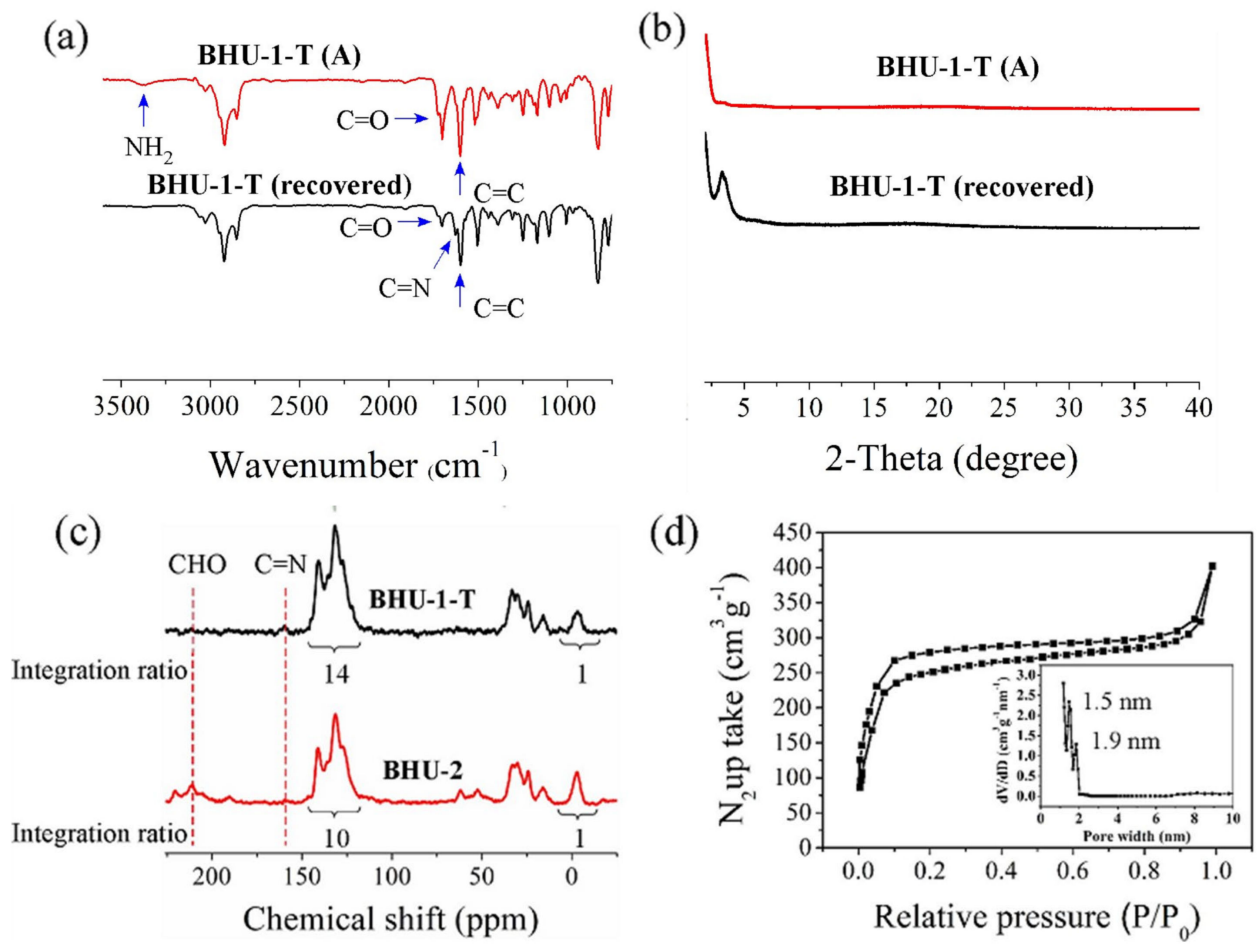
(**a**) IR spectra and (**b**) PXRD patterns of BHU-1-T(A) (above) and BHU-1-T (recovered) after heating. The 1600 and 1700 cm^−1^ peaks were assigned to the aromatic rings and aldehyde groups respectively. The presence of some unreacted aldehyde in the formed COFs was commonly known [28,29,30,31]. (**c**) ^13^C CP-MAS solid-state NMR spectra of BHU-1-T and BHU-2 (the peaks at 50 ppm belong to the methanol solvents trapped with BHU-2). (**d**) Nitrogen adsorption–desorption isotherms and pore-size distribution of BHU-2.

First, we found that amorphous BHU-1-T(A), a network structure formed by C=C double, was obtained if the tying was performed in wet THF, due to hydrolysis of the imine bonds during tying. In fact, the almost absence of the 1624 cm^−1^ for C=N bonds in the IR spectroscopy of BHU-1-T(A) and the appearance of the broad peaks at 1700 and 3470 cm^−1^ for aldehyde and amine groups respectively (Figure 4a, top) indicated the hydrolysis of imine bonds back to aldehyde and 1,3,5-tri(4-aminophenyl)benzene, the latter of which was trapped within the framework of the interconnected aldehyde subunits. Interestingly, the crystallinity could be recovered if the amorphous BHU-1-T(A) (19 mg/mL) was heated in toluene with 3M aqueous AcOH (6 eq. per imine) at 120 °C for one week as shown in Figure 4b. We reasoned that with the catalysis of AcOH, the hydrolyzed 1,3,5-tri(4-aminophenyl)benzene re-formed the imine bonds with the exposed aldehyde groups on the framework to recover crystallinity. As shown in Figure 4a, the characteristic peak of 1624 cm^−1^ for imine bond [27] re-appeared in BHU-1-T (recovered), and the peaks for aldehyde (1700 cm^−1^) and amines (3470 cm^−1^) were significantly reduced. The lower diffraction intensity in the recovered one was probably caused by partial loss of 1,3,5-tri(4-aminophenyl)benzene into the THF solution during tying.

Although the amorphous-to-crystalline transformation was previously known for COFs, [32,33] the amorphous state in previous work could only be regarded as the intermediate state for the final crystalline COFs. Additionally, these transformations were not reversible because of the formation of permanent and stable bonds. The recovery of crystallinity in our case represented a different and versatile approach because of kept dynamic bonds in the final crystalline state, and this transformation could be utilized to introduce other amine groups into BHU-1-T. 

### 3.5. Hydrolysis of BHU-1-T (BHU-2)

BHU-1-T could be completely hydrolyzed in 1 mL aqueous HCl (5 M) and 9 mL THF solution for 16 h at room temperature to fully remove 1,3,5-tri(4-aminophenyl)benzene groups to generate a gray powder as BHU-2, which was a network structure formed by C=C double. In contrast, BHU-1 completely dissolved in the same acidic solution after 16 h at room temperature. As shown in Figure 4c, solid-state ^13^C-NMR analysis of BHU-2 indicated a complete loss of imine bonds (159 ppm) after hydrolysis, and reappearance of the aldehyde groups. The ratio between integrated peak areas between dimethyl groups on the Si atom (−3 ppm) and aromatic ones (116 ppm–147 ppm) was around 1:14, and this was consistent with the expected value based on the structural model (1:14) as shown in Scheme 2. If the 1,3,5-tri(4-aminophenyl) benzene was fully hydrolyzed and removed, the ratio should be changed to around 1:10, which was actually confirmed by the experimental value (1:10). Moreover, there was almost absence an of the 1624 cm^−1^ for C=N bonds in the IR spectroscopy of BHU-2. (Figure S4b). XPS revealed the absence of any N peak in BHU-2, again supporting the complete hydrolysis and removal of the triphenylamine groups. The full removal of 1,3,5-tri(4-aminophenyl) benzene should permit more space within the framework and also additional flexibility of the framework. As shown in Figure 4d, the permanent porosity was estimated by nitrogen adsorption isotherms with the BET surface increased to 992 m^2^·g^−1^ from 259 m^2^·g^−^^1^ for untied BHU-1, owing to the alkene cross-linking and the absence of triamine ligand. Additionally, the removal of the 1,3,5-tri(4-aminophenyl) benzene groups generated a second kind of pore, with diameters of 1.5 nm and 1.9 nm (Figure 4d). It had to be noted that BHU-2 became completely amorphous after full hydrolysis, and this was probably due to the flexible nature of the alkyl chains in comparison to the rigid 1,3,5-tri(4-aminophenyl)benzene.(Figure S7) Moreover, the enhanced BET surface indicated the most desired properties of COF were maintained, and BHU-2 actually belonged to the family of porous organic materials, which had been intensively pursued for various applications [34].

### 3.6. Hydrosilylation of Styrene Catalyzed by Pt@BHU-2 or Pt@BHU-2-Oxidate

The hydrosilylation reaction is one of the most important ways of forming silicon-carbon bonds, which could be catalyzed by radical- [35,36] or transition metal-mediated processes [37,38]. Compared with homogeneous catalysts, heterogeneous catalysts can be recovered from reaction mixtures and reused, reducing the economic and environmental costs. Several platinum-based heterogeneous catalysts have been reported for the hydrosilylation of alkenes or alkynes, however, currently, most heterogeneous catalysis still have the disadvantages of low efficiency and leaching of active substances [39,40,41].

**Figure 5:**
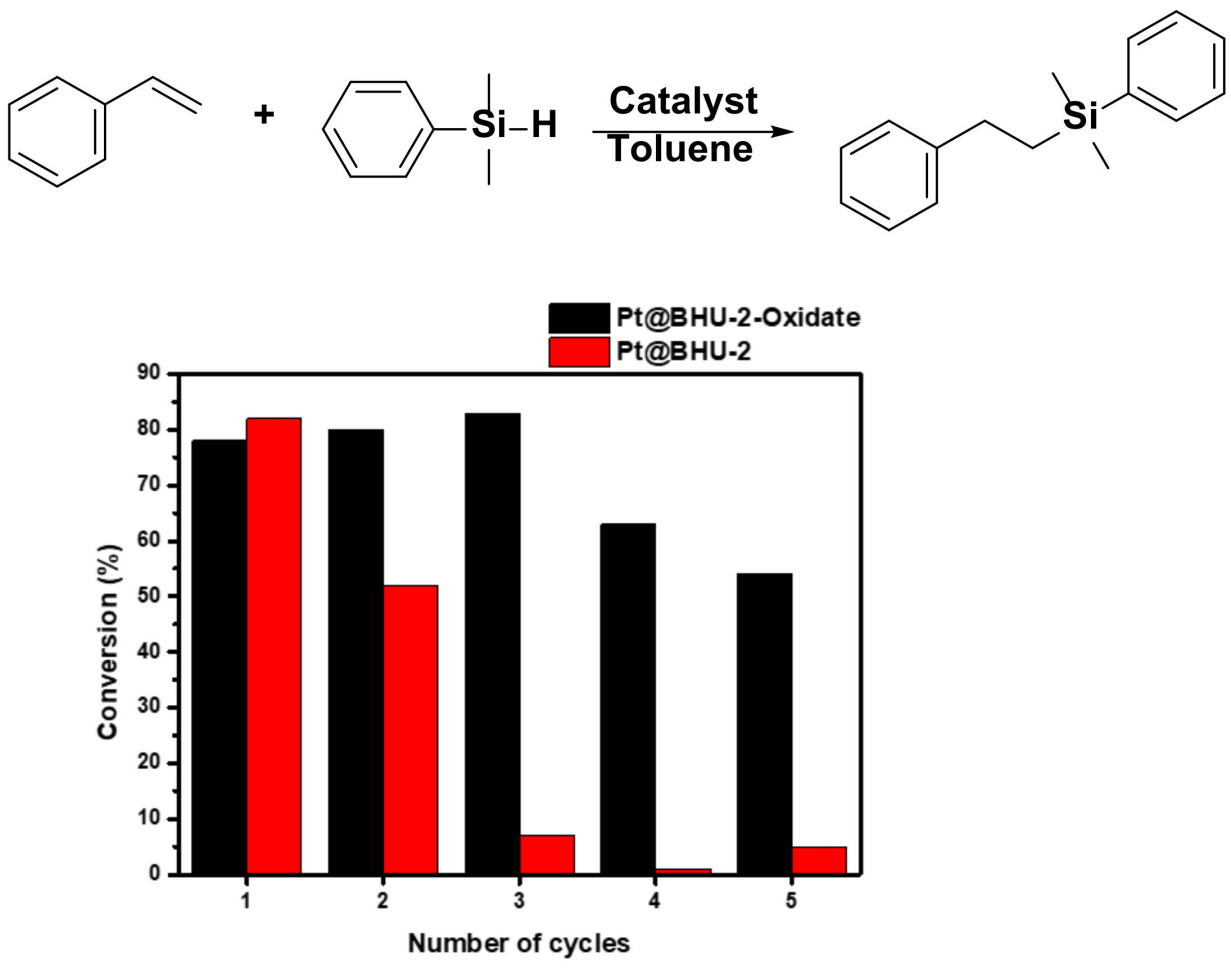
Recycling investigations of Pt@BHU-2 and Pt@BHU-2-Oxidate in the addition reaction between dimethylphenylsilane and styrene. Notice that 4 and 5 can achieve the same conversion rate for 13 h. Reaction conditions: styrene (10.42 mg, 0.1 mmol), dimethylphenylsilane (27.25 mg, 0.2 mmol) catalyst (1 mg, Pt: 0.000957 mg(Pt@BHU-2); 0.00127 mg(Pt@BHU-2-Oxidate), toluene (1 mL), temperature (60 ^o^C), time (8 h), and air.

**Figure 6:**
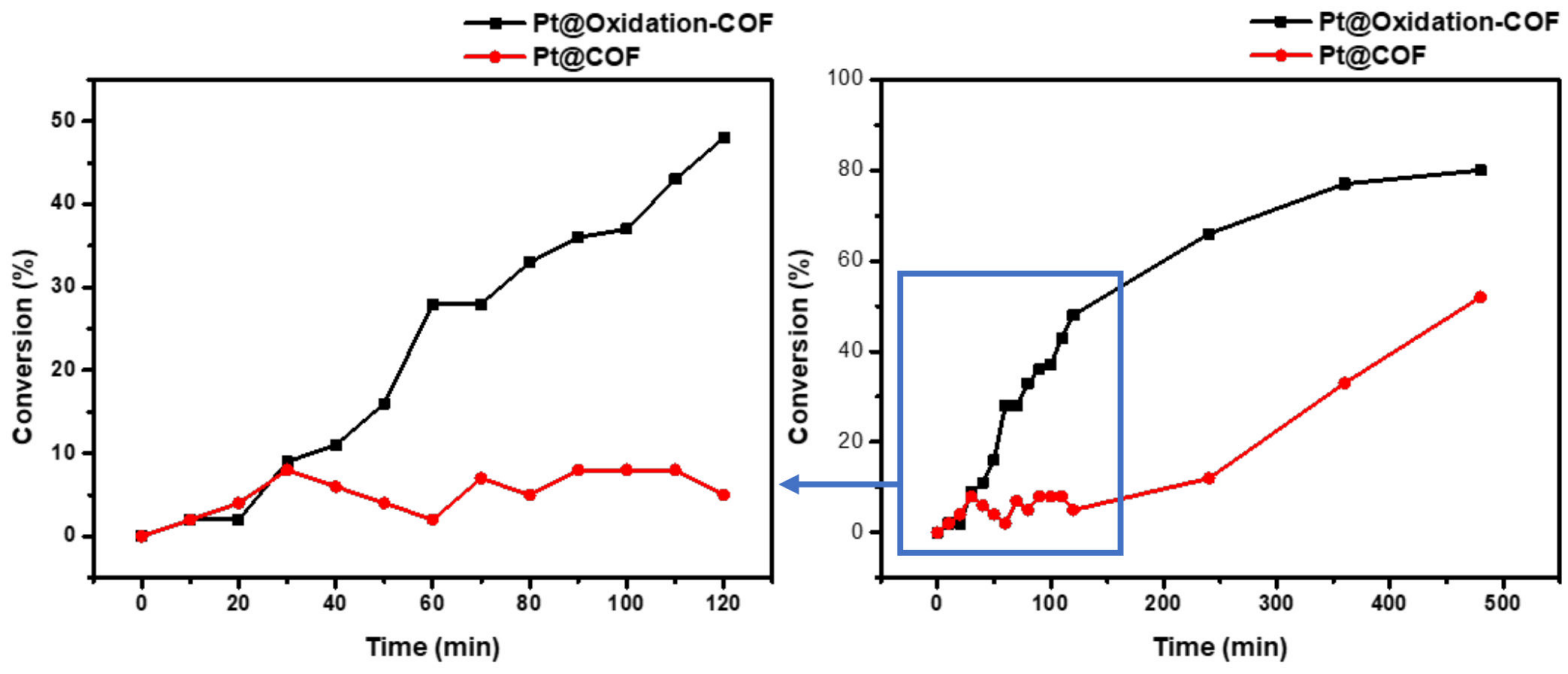
Catalytic activity investigations of Pt@BHU-2 and Pt@BHU-2-Oxidate in the addition reaction between dimethylphenylsilane and styrene. Reaction conditions: styrene (10.42 mg, 0.1 mmol), dimethylphenylsilane (27.25 mg, 0.2 mmol) catalyst (1 mg), toluene (1 mL), temperature (60 ^o^C), time (8 h), and air.

In fact, treatment of BHU-2 with Na_2_PtCl_4_ led to the generation of Pt@BHU-2. The microscopic features of the samples were characterized by a field emission scanning electron microscope (FE-SEM) coupled with an energy-dispersive X-ray spectroscope (EDS) (as shown in Figure S8). The corresponding EDS elemental mapping could prove platinum is present in Pt@BHU-2. The results of ICP-MS showed 0.0957% (wt%) Pt loading on Pt@BHU-2 and 0.127% (wt%) Pt loading on Pt@BHU-2-Oxidate. According to the results of ICP-MS, we could estimate that Pt@BHU-2 showed a TOF of 2089^−1^ for the reaction between styrene and dimethylphenylsilane, as shown in Figure 5. The catalyst could be filtered and reused for the fifth run although the TOF was lowered to 152 h^−1^ (Figure 5 and Figure 6). Interestingly, both the activity and the recyclability could be improved by oxidation of the aldehyde groups to carboxylic acid in BHU-2 by sodium chlorite (BHU-2-Oxidate). Then we performed nitrogen adsorption–desorption isotherms of BHU-2-Oxidate (Figure S9). As shown in Figure S10, platinum was present in Pt@BHU-2-Oxidate. Meanwhile, the appearance of the peaks at 1690 cm^−1^ for C=O bonds of carboxylic acid indicated the conversion of aldehyde to carboxylic acid. (Figure S11) [42]. Based on these results, we could infer that Pt@BHU-2-Oxidate was successfully prepared. In Figure 6, we found Pt@BHU-2-Oxidate had the highest catalytic activity in the first 2 h, implying that carboxylic acid promoted the experimental process. The positive role of the carboxylic acid is consistent with previous reports [43,44]. Meanwhile, the formed Pt@BHU-2-Oxidate showed a TOF of 1497 h^−1^ (Figure 5). A total of 69% of the original activity was maintained during the fifth run. Compared with that, Pagliaro’s group reported a new SiliaCat Pt(0) catalyst which could be effectively used for hydrosilylation reactions, whereas the conversion decreased after the second run [45]. In 2019, A novel SBA-15-supported platinum catalyst with naphthalenolimine and COD binary ligands was conveniently prepared by Hu’s group. The catalyst exhibits a good conversion rate during the first three cycles. However, it dropped to 30.1% at the fourth [46]. In 2020, Tait’s group had prepared a series of heterogeneous Pt catalysts. Although these catalysts have high activity, the activity of the catalysts decreases significantly after several cycles. The best performing platinum catalyst also showed a reduction in activity to 37.7% of the initial activity after 4 cycles [47]. Given the high cost of Pt, the good recycling properties of BHU-2-Oxidate are attractive.

## 4. Conclusions

Overall, the main theme of this work is to utilize a bond formation event that is both reversible and dynamic to permit good crystallinity, to construct the framework, and then to trigger the occurrence of the second formation event, not reversible but permanent, to tie up the framework. With this idea, we demonstrate in this manuscript that alkene metathesis within the covalent organic framework help permanently cross-link internal subunits and therefore serves to significantly increase framework stability. The obtained stable framework allowed the recovery of crystallinity and complete removal of the 1,3,5-tri(4-aminophenyl)benzene subunits for enhanced porosity, and all these significantly increased the versatility of stable COFs. The realization of these can be challenging for other approaches. In addition, the use of tied COF for stable heterogeneous Pt catalyst support demonstrated the practical application of our approach. 

Given the simplicity of the reaction to prepare the precursor of the *C_3_* symmetrical aldehyde compound, it is expected that a variety of similar COFs can be prepared with high stability in the future. For example, similar *C_3_* symmetrical amine can be synthesized using the method just by using relative 4-aminophenylboronic acid, and eventually, the amine group decorated framework can be generated. The presence of amine will generate opportunities to decorate the free space through various efficient reactions. 

In addition, the same strategy can be applied to other kinds of framework materials, such as MOFs, a lot of which are constructed by *C_3_* symmetrical organic precursors [48]. For example, the substitution of the 4-aldehydephenylboronic acid with 4-carboxyphenylboronic acid during the synthesis of L1 will lead to the synthesis of a *C_3_* symmetrical carboxylic acid molecule. As already known, *C_3_* carboxylates have been investigated extensively with different metal ions to form a rich library of various frameworks, including some extremely porous ones, [49] but most of them suffer low stability especially in acidic/basic conditions. It is expected that the hierarchical bond formation, as demonstrated in this work, can serve to enhance the stabilities of the already known frameworks by *C_3_* symmetrical carboxylates by use of the alkene containing a version of *C_3_* carboxylates. In addition, removal of the metal ions in tied MOFs will lead to COFs with porous frameworks internally decorated by carboxylate or carboxylic acid, the realization of which has been daunting due to the strong coordination effect and high reactivity of these functional groups.

## Data Availability

The data presented in this study are available on request from the correspondent author.

## Supplementary Materials

The following supporting information can be downloaded at: https://www.mdpi.com/article/10.3390/nano12030499/s1, Figure S1: 1H-NMR of L1; Figure S2: Pore-size distribution of BHU-1; Figure S3: AB stacking structure of BHU-1 with the unit cell axis indicated; Figure S4: (a) IR spectra of BHU-1-T, and b) IR spectra of BHU-1-T and BHU-2; Figure S5: Pore-size distribution of BHU-1-T; Figure S6: TGA of BHU-1, BHU-1-T, BHU-2, and BHU-2-Oxidate; Figure S7: (a) Pore structure of BHU-1. (b) Pore structure of BHU-1-T. (c) Pore structure of BHU-2. The blue and black layer stands for the next two layers; Figure S8: SEM images of Pt@BHU-2 and corresponding EDS elemental mapping (C, O, and Pt) of Pt@BHU-2; Figure S9: (a) Nitrogen adsorption-desorption isotherms (b) and pore-size distribution of BHU-2-Oxidate Figure S10: SEM images of Pt@BHU-2-Oxidate and corresponding EDS elemental mapping (C, O, and Pt) of Pt@BHU-2-Oxidate; Figure S11: IR spectra of BHU-2 and BHU-2-Oxidate; Table S1: Parameters for the simulated AA stacking crystal structure of BHU-1; Table S2: Parameters for the simulated AB stacking crystal structure of BHU-1.
