# Supplementary material for: Tying Covalent Organic Frameworks through Alkene Metathesis and Supported Platinum as Efficient Catalysts for Hydrosilylation
Source: Nanomaterials (Basel). 2022 Jan 31;12(3):499. doi: 10.3390/nano12030499 (PMC8915182; doi:10.3390/nano12030499)
Supplement: Supplementary file 1 [file nanomaterials-12-00499-s001.zip › nanomaterials-1548881 SI.pdf]

Supporting Information

Tying Covalent Organic Frameworks through Alkene Metathesis and Supported Platinum as Efficient Catalysts for Hydrosilylation

Defa Gu [1,+], Guangwen Li [2,+], Yushan Liu [3], Yuzhou Liu [1,4,*]

[1] School of Chemistry, Beihang University, Haidian District, Bejing, 100191, China; gudefa1993@163.com;
[2] Research Institute of Petroleum Processing, SINOPEC, Beijing 100083, China; liguangwen.ripp@sinopec.com;
[3] Trinity School of Durham and Chapel Hill, Durham, NC, 27705, USA; 13611017779@163.com;
[4] Advanced Innovation Center for Biomedical Engineering, Beihang University, Haidian District, Beijing, 100191, China;
* Correspondence: liuyuzhou@buaa.edu.cn (Y.L.); Tel.: +86–010-82316866; Fax: +86–010-82316866;
+ These authors contributed equally to this work.

Citation: Gu.D.; Li.G.; Liu.Y.; Liu.Y,. Tying Covalent Organic Frameworks through Alkene Metathesis and Supported Platinum as Efficient Catalysts for Hydrosilylation . *Nanomaterials* **2022**, *12*, 499. https://doi.org/10.3390/nano12030499

Academic Editor: Takuya Kitaoka

Received: 25 December 2021
Accepted: 29 January 2022
Published: 31 January 2022

Publisher's Note: MDPI stays neutral with regard to jurisdictional claims in published maps and institutional affiliations.

Copyright: © 2022 by the authors. Submitted for possible open access publication under the terms and conditions of the Creative Commons Attribution (CC BY) license (https://creativecommons.org/licenses/by/4.0/).

Abstract: Recently there has been a great interest in covalent organic frameworks due to their fascinating properties. Current approaches to improve their hydrolytic stability mainly rely on transformation of the dynamic bonds into strong and irreversible bonds, but these approaches also reduce the versatility of the frameworks. Herein, we would like to demonstrate a way to solve this dilemma by generating highly stable COFs through hierarchical bond formation via olefin metathesis. Our approach allows unprecedented opportunities for post-modification of the inner space through the dynamic imine bonds while maintaining the integrity of the framework. Specifically, we demonstrate an amorphous-to-crystalline transformation. In addition, the porosity can be enhanced by up to 70% after full removal of the amine subunits. Overall, our work provides a new direction for the generation of highly stable while still versatile COFs. Meanwhile, platinum(II) complexes can be supported on **BHU-2 (Pt@BHU-2)** or **BHU-2-Oxidate(Pt@BHU-2-Oxidate)** as efficient catalysts for hydrosilylation.

Experimental Procedures

S1. Materials and Methods

Materials: Unless otherwise mentioned, all chemicals were purchased through InnoChem, Inc. China. THF and Et_2O were distilled from Na/benzophenone. Reactions were monitored using analytical thin layer chromatography. Flash chromatography was performed using silica gel (200-300 mesh).

Characterization and Measurements: Proton nuclear magnetic resonance (^1H-NMR) spectra were recorded on a Bruker Avance 300 and 400 MHz spectrometers using DSMO-d_6 or $CDCl_3$ as the solvent. The ^1H-NMR were referenced to residual solvent signals at 7.26 ppm ($CHCl_3$) or 2.5 ppm (DMSO). Solid state ^{13}C-NMR spectra were collected on JNM-ECZ600R. The matrix-assisted laser desorption ionization time-of-flight (MALDI-TOF) mass spectrum was collected on a Bruker Autoflex III at Tsinghua University. Gas adsorption isotherm was measured on Quadrasorb SI-MP surface area analyzer. FTIR spectra was collected using a Thermo Nicolet Nexus470 FTIR instrument. The crystalline phase structure of all samples were observed on a Bruker-D8-Focus powder diffractometer with (Cu Kα irradiation λ = 0.15406 nm) in the range 2-40° (2θ). Inductively coupled plasma massspectrometry (ICP-MS) was collected on a Aglient 7800. GC-MS was collected on a SHIMADZU GCMS-QP2010 SE. The morphology and composition of samples were characterized on a field emission scanning electron microscope (FE-SEM, JEOL JSM-7500F) coupled with an energy-dispersive X-ray spectroscope(EDS).

S2. Characterization

Compound **L1**: white solid, ^1H-NMR (400 MHz, $CDCl_3$) δ 9.97 (s, 3H), 7.82 (d, *J* = 8.3 Hz, 6H), 7.53 (d, *J* = 8.2 Hz, 6H), 7.13 (d, *J* = 8.3 Hz, 6H), 6.99 (d, *J* = 7.9 Hz, 6H), 6.91 (d, *J* = 8.3 Hz, 6H), 6.83 (d, *J* = 7.9 Hz, 6H), 5.73 (dd, *J* = 17.0, 10.2 Hz, 3H), 4.92 (dd, *J* = 23.7, 6.2 Hz, 6H), 1.92 (d, *J* = 7.5 Hz, 6H), 1.29-1.17 (m, 6H), 1.09 (s, 18H), 0.58-0.43 (m, 6H), 0.05 (s, 18H). MS (MALDI-TOF) (CHCA): *m/z* [m + Na] calcd. 1375, found 1375.

S3. ^1H-NMR spectra

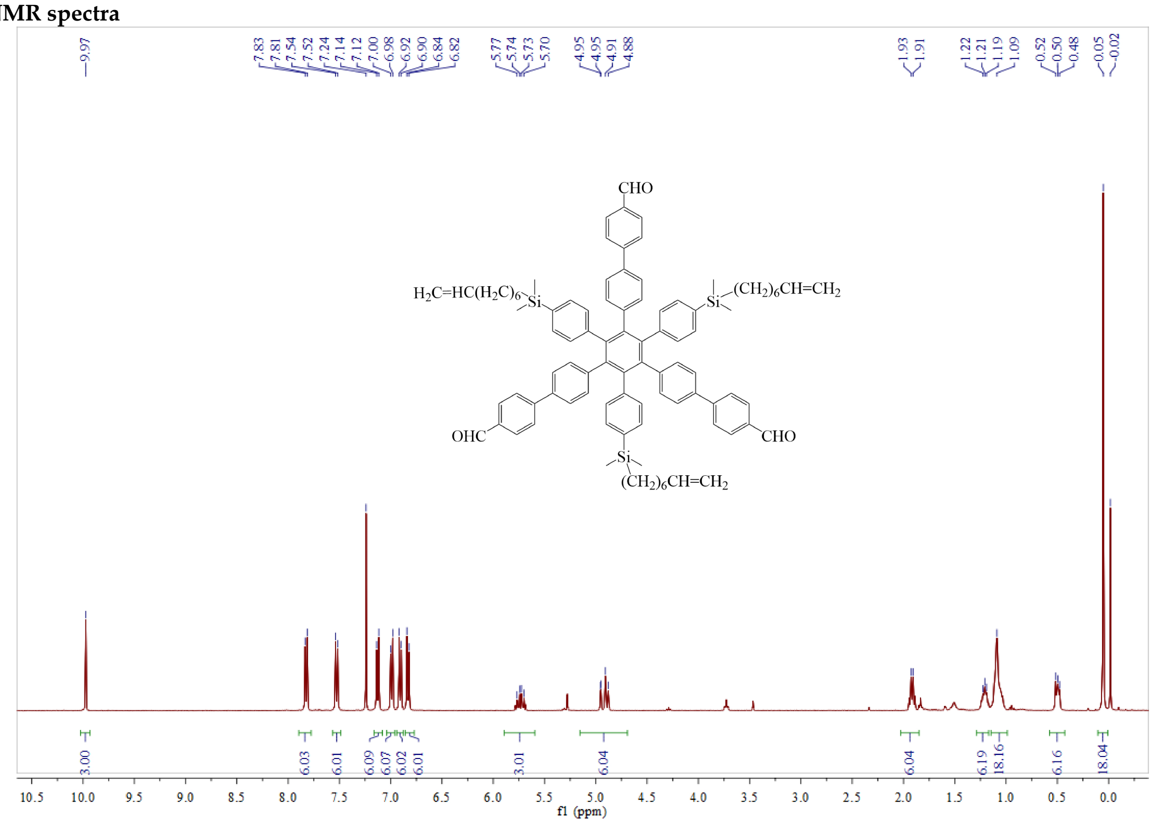

Figure S1. ^1H-NMR of **L1**

S4. Pore-size distribution of BHU-1

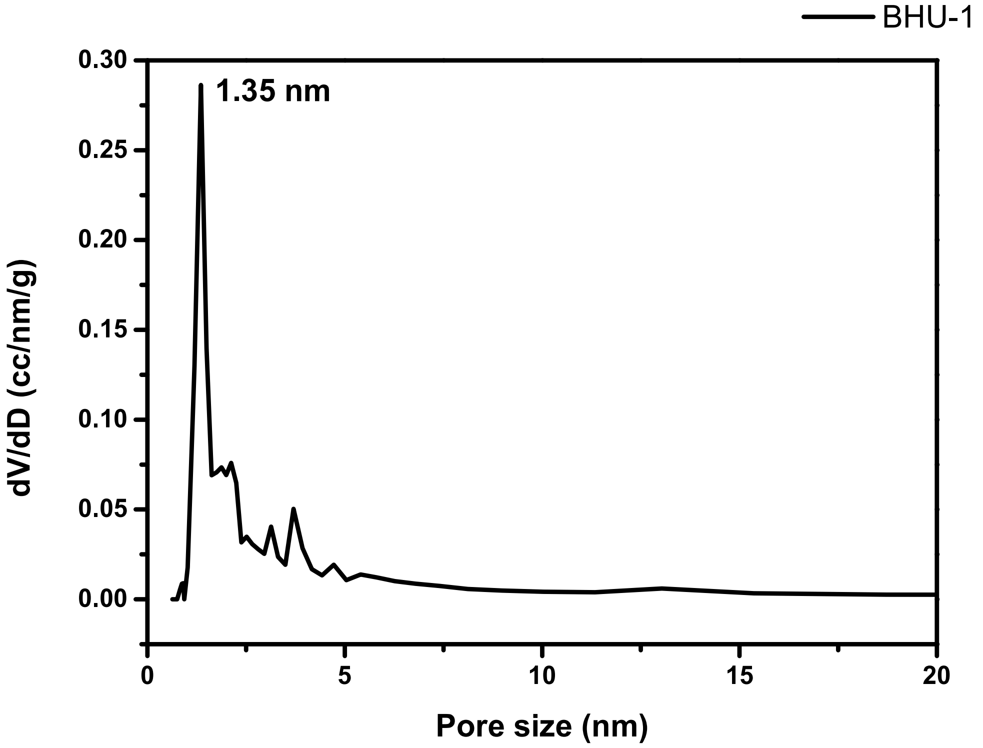

Figure S2. Pore-size distribution of **BHU-1**

S5. Simulation details of the structure model of BHU-1

Geometrical optimization was performed through molecular mechanics approach with the Forcite model of Material Studio Software 6.0 based on the universal force field (UFF). [2,3] The optimized structure is converged to below 10^{-6} kcal/mol in total energy, less than 0.01 eV/Å in atom forces and smaller than 10^{-6} angstrom. Cell parameters are subject to optimize during the process.

Table S1. Parameters for the simulated AA stacking crystal structure of **BHU-1**.

Hexagonal P3<br>a = b = 30.5 A°, c = 5 A°<br>α=β=90° γ=120°			
C	0.43811	0.79849	-0.59848
C	0.42977	0.75737	-0.76449
C	0.47037	0.76427	-0.93191
C	0.51583	0.81131	-0.94755
C	0.5215	0.85292	-0.79739
C	0.48289	0.84559	-0.61713
N	0.55805	0.9014	-0.83953
C	0.60895	0.93056	-0.85372
C	0.79407	0.93525	-0.68855
C	0.80573	0.89732	-0.62526
C	0.85632	0.9104	-0.57006
C	0.89742	0.9612	-0.54656
C	0.8855	0.9955	-0.67349
C	0.83555	0.98434	-0.72754

C	0.97964	1.03234	-0.35498
C	0.94683	0.97942	-0.42091
H	0.5435	0.81566	-1.07759
H	0.4866	0.87496	-0.50133
H	0.62008	0.96718	-0.90402
H	0.77769	0.8602	-0.61725
H	0.86229	0.88073	-0.55337
C	0.38188	0.71206	-0.76591
C	0.37762	0.66368	-0.76768
H	0.41114	0.79487	-0.46439
C	0.96202	1.0631	0.77733
C	0.9961	1.10926	0.90526
C	0.98523	1.14877	0.94174
C	0.93671	1.14098	0.88023
C	0.89832	1.09041	0.84075
C	0.91126	1.05261	0.80245
Si	1.26178	1.07345	0.80604
C	1.27514	1.02699	0.90634
C	1.26254	0.98023	0.72641
C	1.30802	0.98362	0.56668
C	1.30587	0.93225	0.51617
C	1.31137	1.13306	0.87656
C	1.25887	1.07373	0.47166
C	1.18245	1.25595	0.28778
C	1.22608	1.26993	0.44452
C	1.27049	1.31656	0.40571
C	1.27293	1.35103	0.20914
C	1.22821	1.33937	0.06809
C	1.18335	1.29233	0.10787
H	0.46664	0.7354	-1.05108
H	0.91372	1.03161	-0.70729
H	0.41002	0.66158	-0.77084
H	1.01406	1.18354	0.99689
H	0.861	1.08047	0.82851
H	0.88187	1.0167	0.79148
H	1.2541	1.0107	1.08823
H	1.31501	1.04587	0.96244
H	1.2311	0.97141	0.59143
H	1.24903	0.94761	0.85889
H	1.3434	1.00753	0.67344
H	1.31105	1.0019	0.37564
H	1.31411	0.91965	0.70466
H	1.33637	0.93939	0.37669
H	1.34642	1.13642	0.79995
H	1.31629	1.14127	1.08846
H	1.30597	1.16204	0.77986
H	1.25496	1.10561	0.40448
H	1.22569	1.039	0.40672
H	1.29361	1.07675	0.39448
H	1.22633	1.24571	0.58579
H	1.30162	1.3244	0.51865
H	1.22846	1.36385	-0.07221

H	1.15188	1.28413	-0.00221
H	0.82962	1.01283	-0.79112
C	1.11129	2.36628	0.40438
H	1.09665	2.33865	0.24286
H	1.12485	2.35092	0.56081
C	1.15675	2.41572	0.29387
H	1.14368	2.42939	0.12676
H	1.1847	2.40689	0.21318
C	1.18145	2.45441	0.49818
H	1.19982	2.44802	0.64319
C	1.18047	2.49779	0.50334
H	1.16246	2.50584	0.36369
H	1.1978	2.52268	0.64879
H	1.03192	1.11681	0.94431

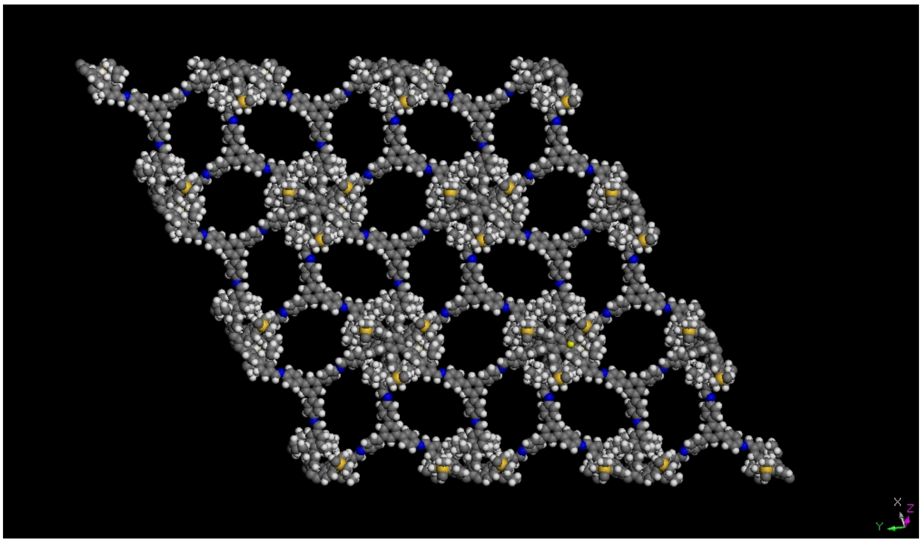

Figure S3. AB stacking structure of **BHU-1** with the unit cell axis indicated.

Table S2. Parameters for the simulated AB stacking crystal structure of **BHU-1.**

Hexagonal P-3<br>a = b = 33.03 A°, c = 12.80 A°			
C	0.38287	0.68603	0.43211
C	0.36295	0.71528	0.43285
C	0.43198	0.70538	0.42698
C	0.452	0.68804	0.35447
C	0.50075	0.70813	0.34573
C	0.53119	0.74648	0.4091
C	0.51147	0.76348	0.48253
C	0.46265	0.74317	0.4917
N	0.57851	0.76918	0.40254
C	0.60721	0.75898	0.34722
C	0.9505	0.97159	0.27129
C	0.97892	0.95038	0.26608
H	0.38457	0.75081	0.43252
H	0.43123	0.66088	0.30581
H	0.4497	0.75639	0.54624
H	0.59304	0.72856	0.30533

H	0.5135	0.69469	0.29157
H	0.53262	0.79104	0.52989
C	1.05586	0.95794	0.75819
C	1.05961	0.92587	0.69167
C	1.07778	0.89807	0.72919
C	1.09295	0.90192	0.83405
C	1.09542	0.93889	0.89474
C	1.07627	0.96577	0.85846
Si	1.10336	0.86278	0.89116
C	1.06066	0.8366	0.98558
C	1.06746	0.80673	1.06997
C	1.02413	0.78237	1.14307
C	1.03143	0.75796	1.23891
C	0.98662	0.72779	1.30496
C	0.96107	0.75296	1.34903
C	0.98996	0.7938	1.41283
C	1.00412	0.79124	1.50928
C	1.15844	0.88938	0.9432
C	1.09906	0.82053	0.80693
C	1.0986	1.0563	0.7184
C	1.11942	1.06062	0.61978
C	1.16767	1.09099	0.60577
C	1.19665	1.11729	0.69018
C	1.17538	1.11138	0.78942
C	1.12713	1.08157	0.80354
C	1.24517	1.14861	0.67667
C	1.27294	1.13726	0.61222
C	1.3214	1.16768	0.60235
C	1.34401	1.21075	0.65665
C	1.31618	1.22288	0.71847
C	1.26775	1.1925	0.72799
H	1.04715	0.92102	0.61717
H	1.07826	0.87417	0.67948
H	1.10939	0.9457	0.96809
H	1.07582	0.98989	0.90795
H	1.02755	0.81481	0.94514
H	1.05737	0.86373	1.02685
H	1.0729	0.78001	1.03289
H	1.09832	0.82934	1.11689
H	0.99448	0.75642	1.09753
H	1.01563	0.80849	1.17131
H	1.04432	0.73509	1.2116
H	1.05836	0.78441	1.28911
H	0.96171	0.69854	1.25677
H	0.99605	0.71277	1.37023
H	0.93099	0.72791	1.39507
H	0.947	0.76341	1.28396
H	1.00007	0.82527	1.38308
H	1.02383	0.82018	1.54954
H	0.99522	0.76068	1.54204
H	1.16462	0.8636	0.98209
H	1.16305	0.91596	0.99968

H	1.18417	0.90575	0.88114
H	1.10631	0.79596	0.84812
H	1.06391	0.80136	0.77409
H	1.12432	0.8365	0.74368
H	1.09933	1.04224	0.55731
H	1.1813	1.09409	0.5326
H	1.19491	1.1289	0.85298
H	1.11281	1.07875	0.87585
H	1.25831	1.10625	0.57258
H	1.34	1.15766	0.55565
H	1.33094	1.254	0.75798
H	1.24912	1.20304	0.7731

S6. IR spectroscopy of BHU-1-T and BHU-2

a)

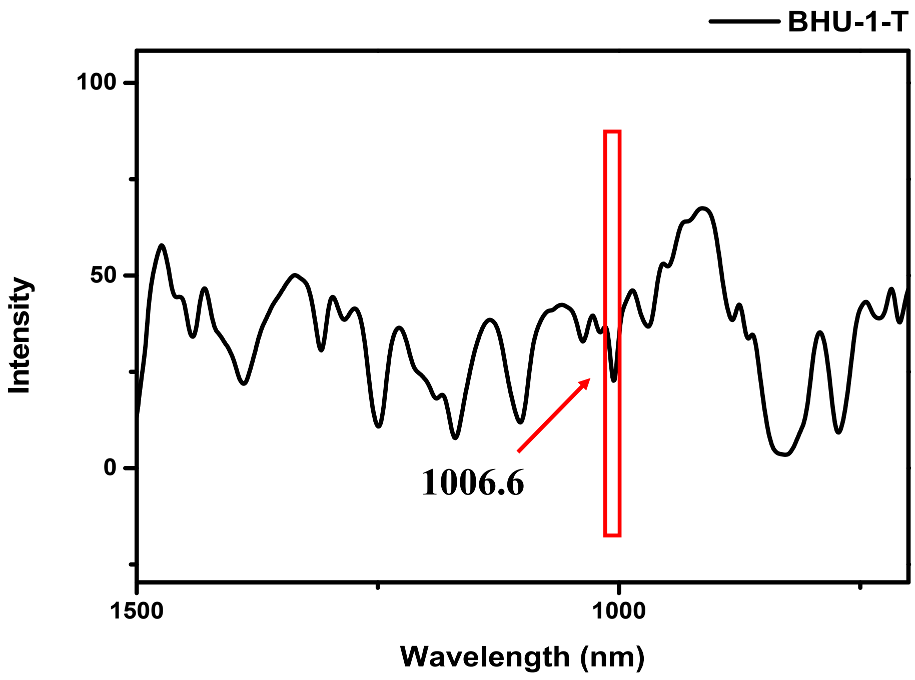

b)

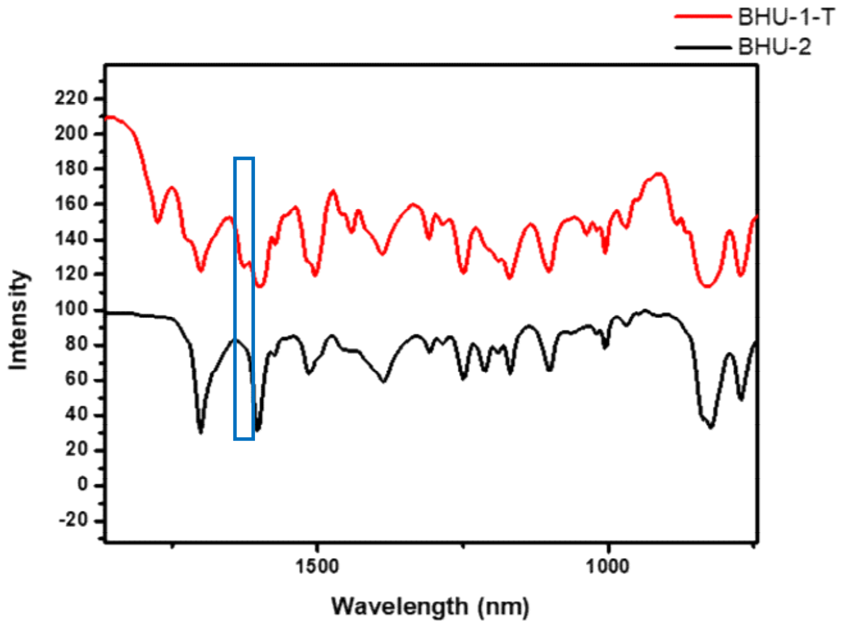

Figure S4. a) IR spectra of **BHU-1-T b)** and IR spectra of **BHU-1-T and BHU-2**

S7. Pore-size distribution of BHU-1-T

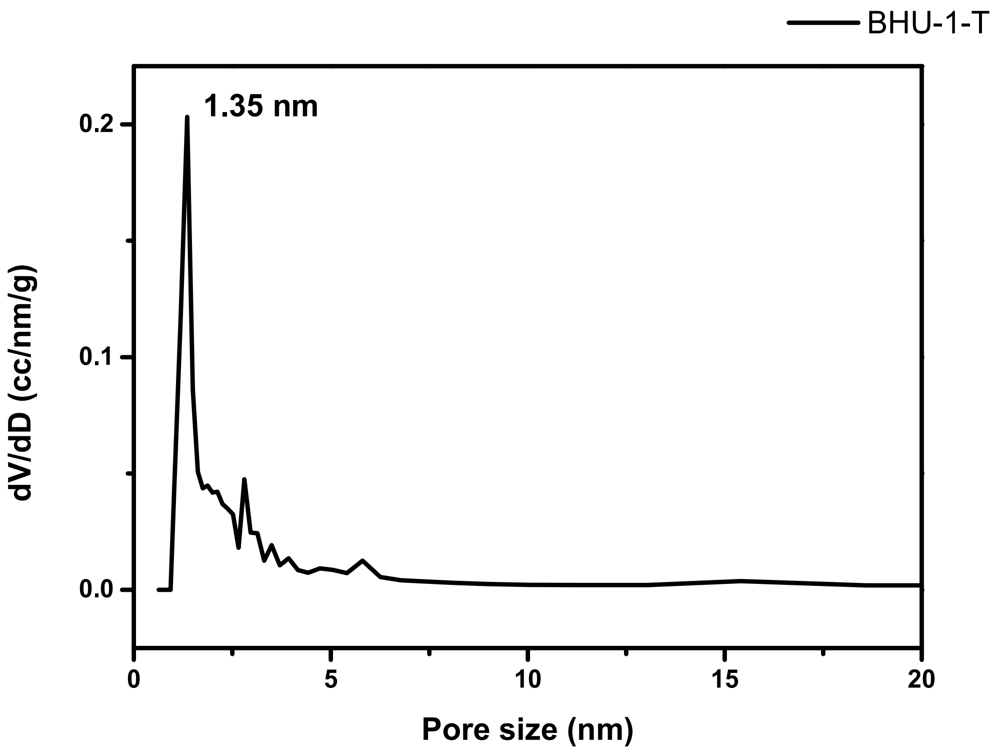

Figure S5. Pore-size distribution of **BHU-1-T**

S8. TGA of BHU-1, BHU-1-T, BHU-2 and BHU-2-Oxidate

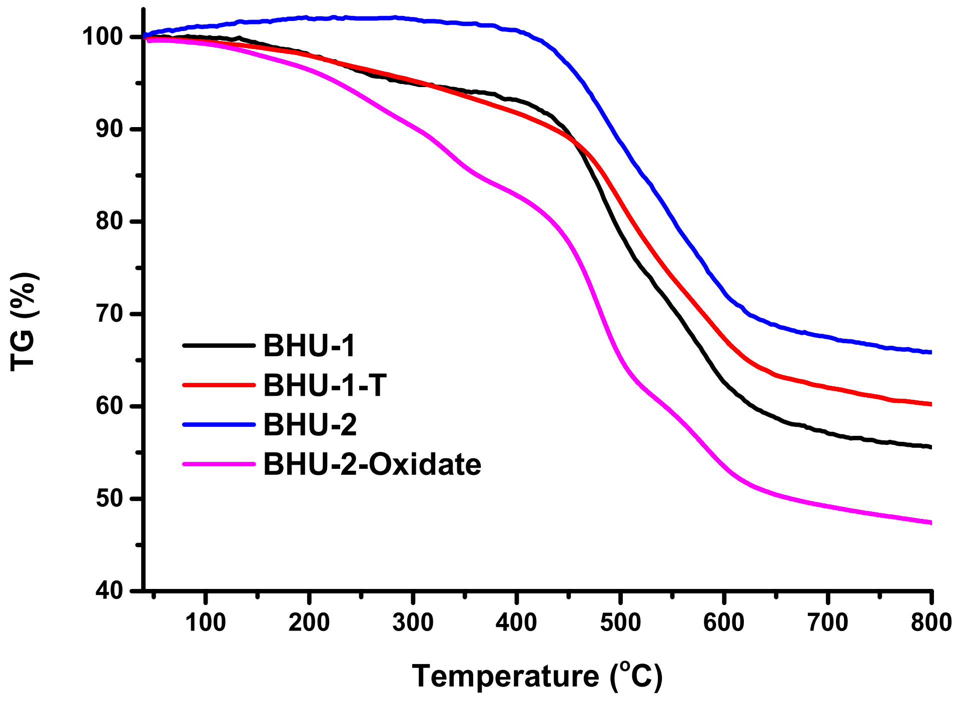

Figure S6. TGA of BHU-1, BHU-1-T, BHU-2 and BHU-2-Oxidate

S9. Pore structure

(a)

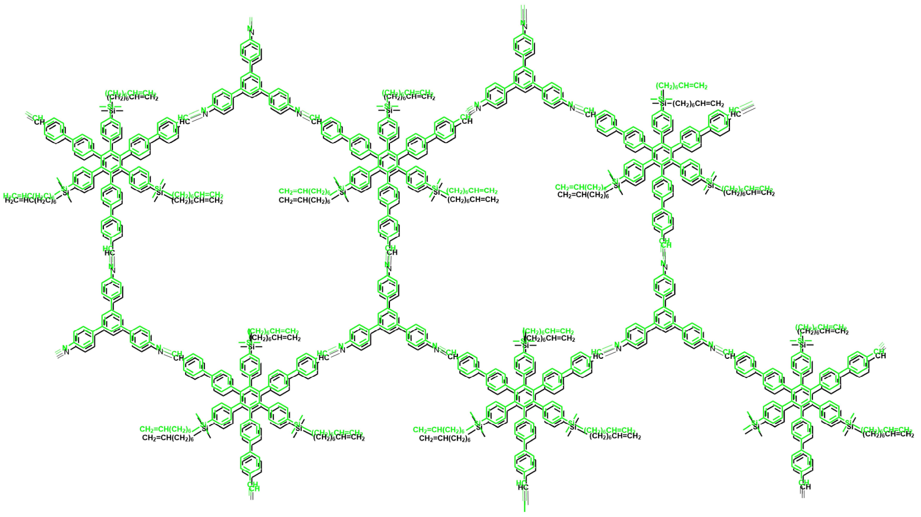

BHU-1

(b)

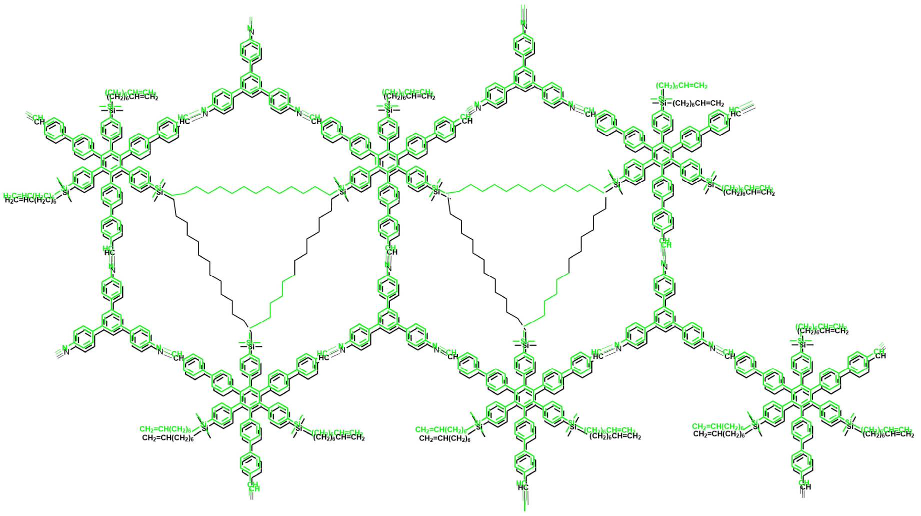

BHU-1-T

(c)

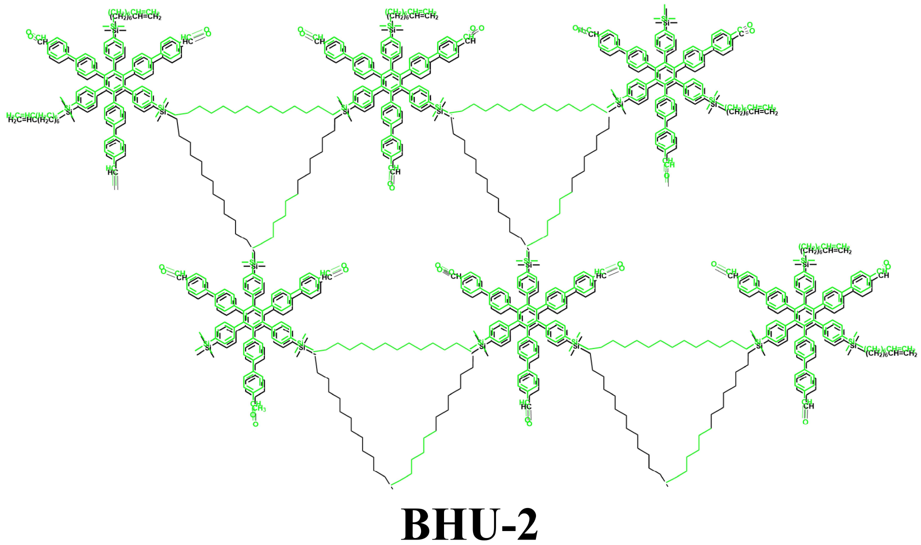

BHU-2

Figure S7. (a) pore structure of **BHU-1**. (b) pore structure of **BHU-1-T**, (c) pore structure of **BHU-2**. The blue and black layer stand for the next two layer.

S10. SEM images of Pt@BHU-2 and corresponding EDS elemental mapping (C, O and Pt) of Pt@BHU-2

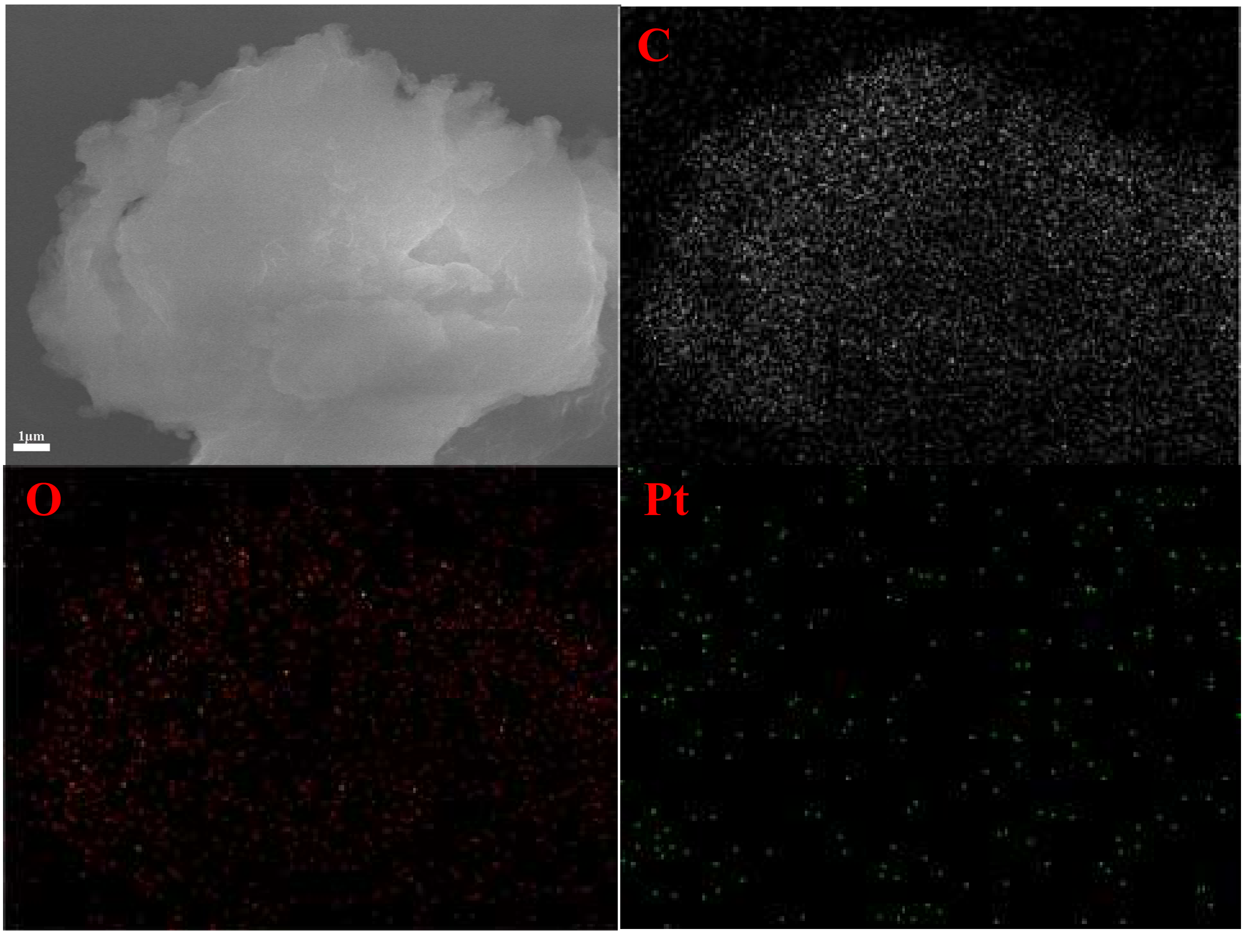

Figure S8. SEM images of **Pt@BHU-2** and corresponding EDS elemental mapping (C, O and Pt) of **Pt@BHU-2**

S11. Nitrogen adsorption-desorption isotherms and pore-size distribution of BHU-2-Oxidate.
a)

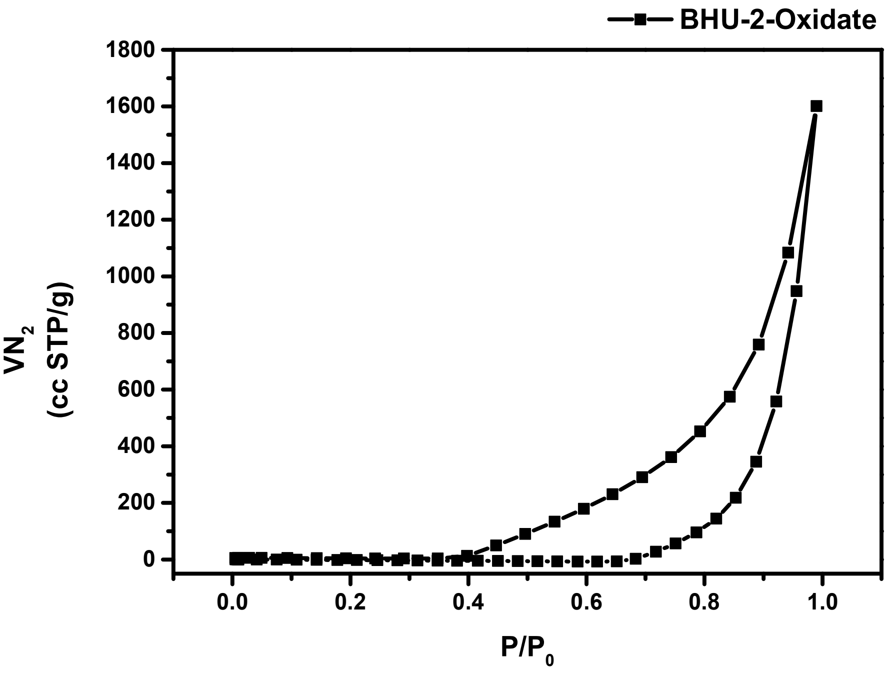

b)

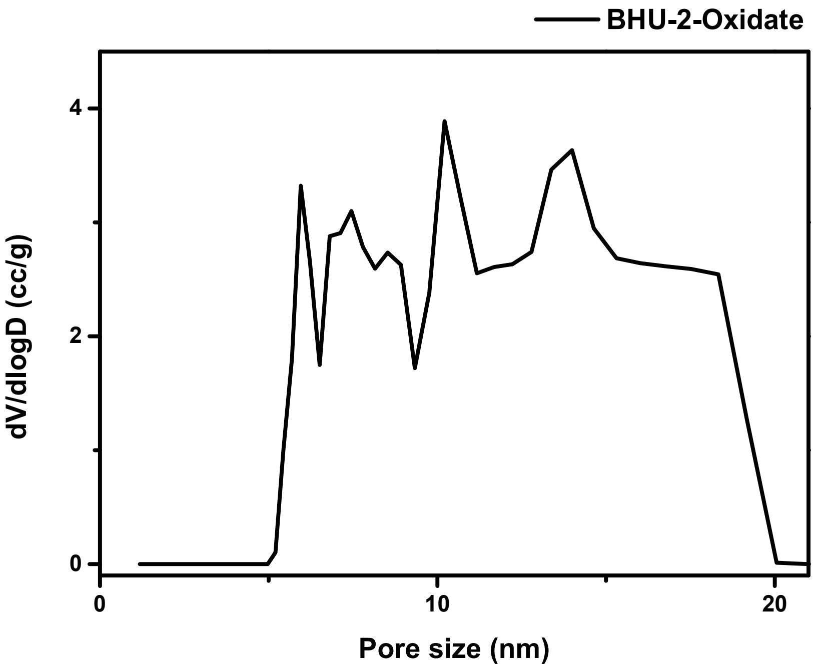

Figure S9. a) Nitrogen adsorption-desorption isotherms b) and pore-size distribution of **BHU-2-Oxidate**

S12. SEM images of Pt@BHU-2-Oxidate and corresponding EDS elemental mapping (C, O and Pt) of Pt@BHU-2-Oxidate

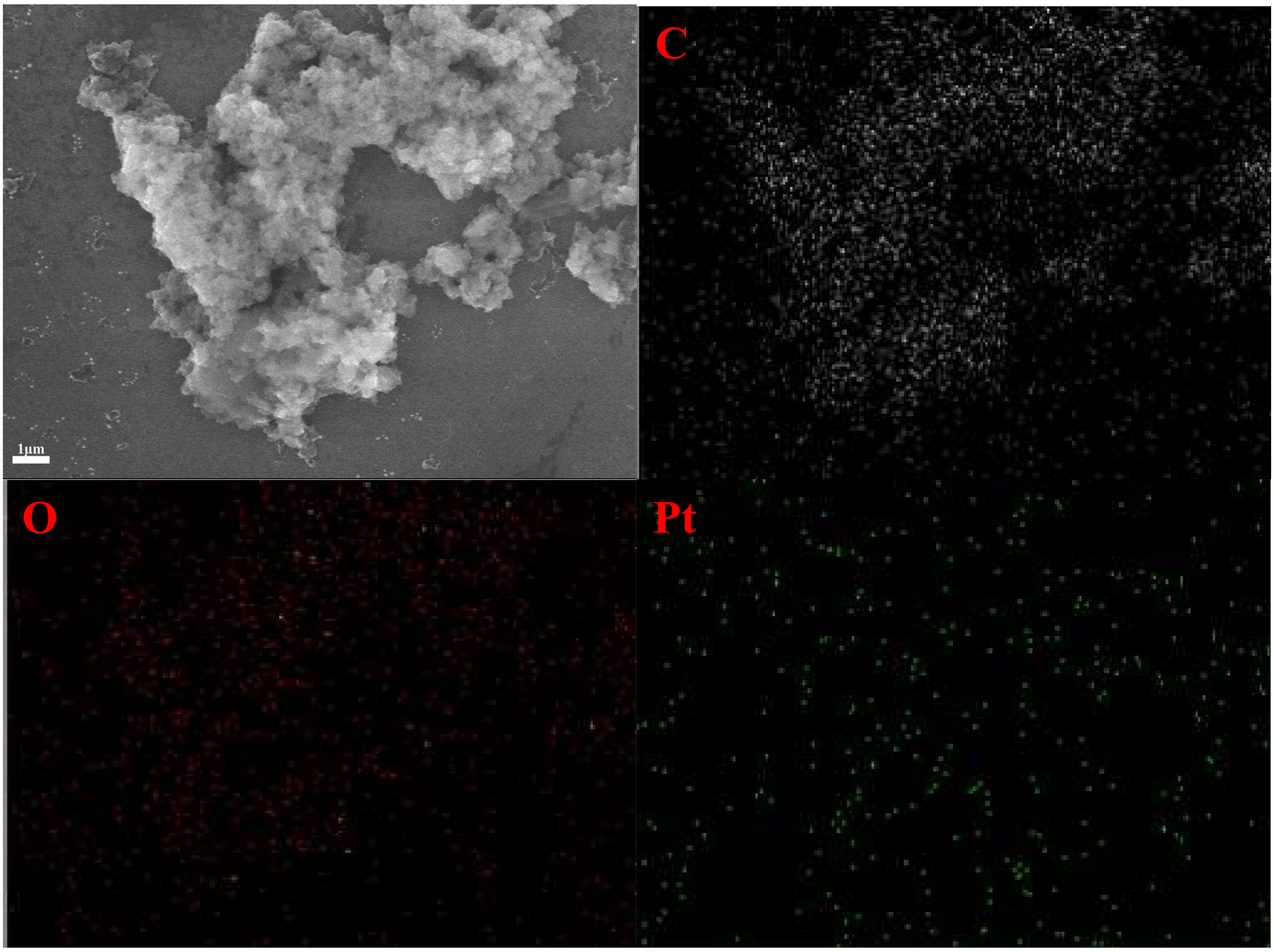

Figure S10. SEM images of **Pt@BHU-2-Oxidate** and corresponding EDS elemental mapping (C, O and Pt) of **Pt@BHU-2-Oxidate**

S13. IR spectra of **BHU-2** and **BHU-2-Oxidate**

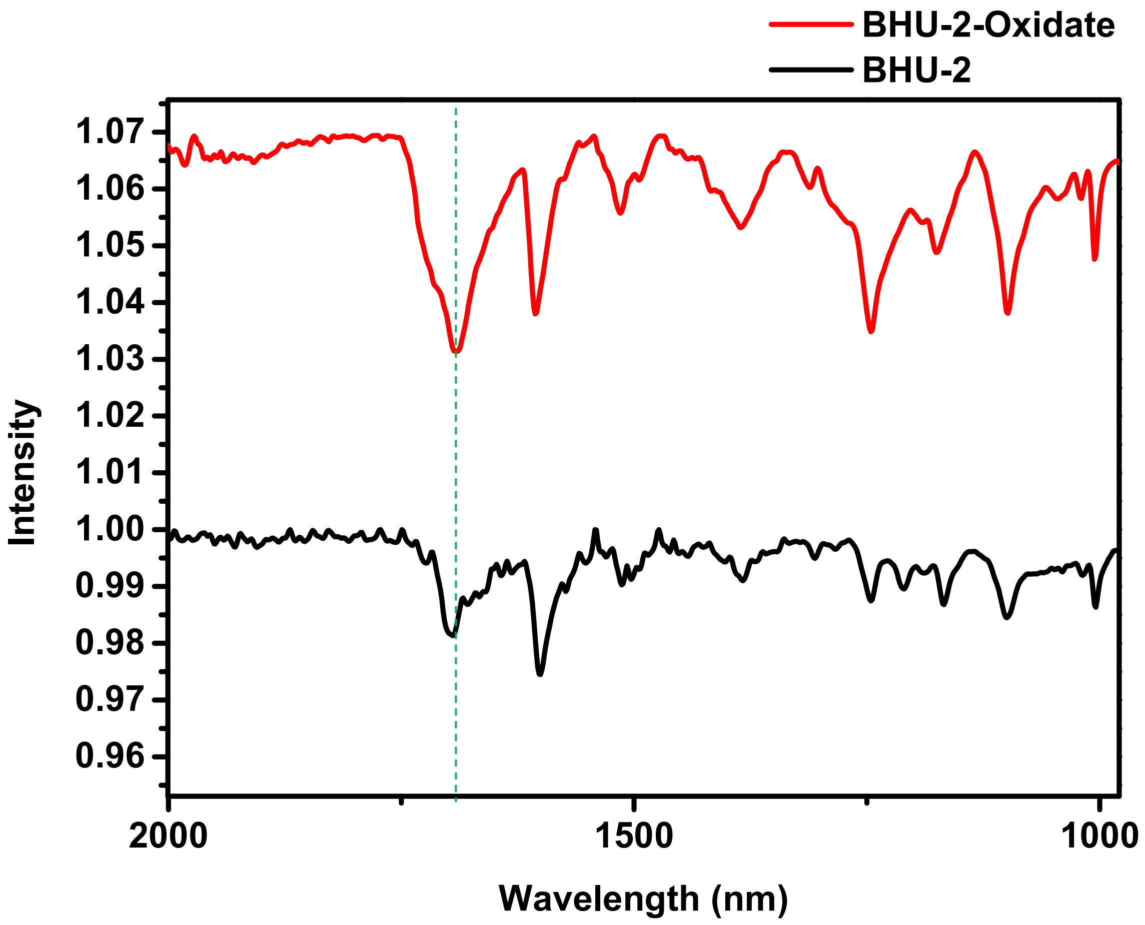

Figure S11. IR spectra of **BHU-2** and **BHU-2-Oxidate**

References

1. Odejinmi, S. I.; Wiemer, D. F.; Synthesis of Arieianal, a Prenylated Benzoic Acid from *Piper arieianum*. *J. Nat. Prod.* **2005,** *68*, 1375-1379.
2. Accelrys, Inc., *Materials Studio, 4.3V*, Accelrys, Inc., San Diego, CA, **2008**.
3. Rappé, A. K.; Casewit, C. J.; Colwell, K. S.; Goddard III, W. A.; Skiff, W. M. UFF, a full periodic table force field for molecular mechanics and molecular dynamics simulations. *J. Am. Chem.Soc.* **1992**, *114*, 10024-10035.

Author Contributions

[+] These authors contributed equally to this work
